# Activation of endothelial NO synthase and P2X7 receptor modification mediates the cholinergic control of ATP-induced interleukin-1β release by mononuclear phagocytes

**DOI:** 10.3389/fimmu.2023.1140592

**Published:** 2023-03-09

**Authors:** Katrin Richter, Nilay Asci, Vijay K. Singh, Sanaria Hawro Yakoob, Marion Meixner, Anna Zakrzewicz, Juliane Liese, Andreas Hecker, Sigrid Wilker, Sabine Stumpf, Klaus-Dieter Schlüter, Marius Rohde, Axel Gödecke, Winfried Padberg, Ivan Manzini, Günther Schmalzing, Veronika Grau

**Affiliations:** ^1^ Laboratory of Experimental Surgery, Department of General and Thoracic Surgery, Justus-Liebig-University Giessen, German Centre for Lung Research (DZL), Cardio Pulmonary Institute (CPI), Giessen, Germany; ^2^ Department of Paediatric Haematology and Oncology, Justus-Liebig-University, Giessen, Germany; ^3^ Institute of Clinical Pharmacology, RWTH Aachen University, Aachen, Germany; ^4^ Institute of Physiology, Justus-Liebig-University Giessen, Giessen, Germany; ^5^ Institute of Cardiovascular Physiology, University Hospital Düsseldorf, Düsseldorf, Germany; ^6^ Department of Animal Physiology and Molecular Biomedicine, Justus-Liebig-University Giessen, Giessen, Germany

**Keywords:** inflammation, P2X7 receptor, CHRNA7, CHRNA9, CHRNA10, endothelial NO synthase, monocytes, macrophages

## Abstract

**Objective:**

The pro-inflammatory cytokine interleukin-1β (IL-1β) plays a central role in host defense against infections. High systemic IL-1β levels, however, promote the pathogenesis of inflammatory disorders. Therefore, mechanisms controlling IL-1β release are of substantial clinical interest. Recently, we identified a cholinergic mechanism inhibiting the ATP-mediated IL-1β release by human monocytes *via* nicotinic acetylcholine receptor (nAChR) subunits α7, α9 and/or α10. We also discovered novel nAChR agonists that trigger this inhibitory function in monocytic cells without eliciting ionotropic functions at conventional nAChRs. Here, we investigate the ion flux-independent signaling pathway that links nAChR activation to the inhibition of the ATP-sensitive P2X7 receptor (P2X7R).

**Methods:**

Different human and murine mononuclear phagocytes were primed with lipopolysaccharide and stimulated with the P2X7R agonist BzATP in the presence or absence of nAChR agonists, endothelial NO synthase (eNOS) inhibitors, and NO donors. IL-1β was measured in cell culture supernatants. Patch-clamp and intracellular Ca^2+^ imaging experiments were performed on HEK cells overexpressing human P2X7R or P2X7R with point mutations at cysteine residues in the cytoplasmic C-terminal domain.

**Results:**

The inhibitory effect of nAChR agonists on the BzATP-induced IL-1β release was reversed in the presence of eNOS inhibitors (L-NIO, L-NAME) as well as in U937 cells after silencing of eNOS expression. In peripheral blood mononuclear leukocytes from eNOS gene-deficient mice, the inhibitory effect of nAChR agonists was absent, suggesting that nAChRs signal *via* eNOS to inhibit the BzATP-induced IL-1β release. Moreover, NO donors (SNAP, S-nitroso-N-acetyl-DL-penicillamine; SIN-1) inhibited the BzATP-induced IL-1β release by mononuclear phagocytes. The BzATP-induced ionotropic activity of the P2X7R was abolished in the presence of SIN-1 in both, *Xenopus laevis* oocytes and HEK cells over-expressing the human P2X7R. This inhibitory effect of SIN-1 was absent in HEK cells expressing P2X7R, in which C377 was mutated to alanine, indicating the importance of C377 for the regulation of the P2X7R function by protein modification.

**Conclusion:**

We provide first evidence that ion flux-independent, metabotropic signaling of monocytic nAChRs involves eNOS activation and P2X7R modification, resulting in an inhibition of ATP signaling and ATP-mediated IL-1β release. This signaling pathway might be an interesting target for the treatment of inflammatory disorders.

## Introduction

Extracellular ATP is sensed by two main classes of purinergic receptors, the G protein-coupled P2Y receptors and the P2X receptors that form trimeric ligand-gated ion channels (1–5). P2X receptors are of outstanding interest as therapeutic targets, as they are expressed by most human or animal cell types and are involved in pain (6) as well as in the pathogenesis of numerous diseases including cancer, cardiovascular disease (7) and virtually all inflammatory diseases (8). The mammalian P2X receptors largely differ in their rates of desensitization and in their affinity towards ATP (5). In both counts, the P2X7 receptor (P2X7R) is an extreme: ATP concentrations in the high micromolar range are needed for P2X7R activation, and there is a complete lack of desensitization (5). In contrast to other P2X receptors, the P2X7R has a characteristic large cytoplasmic C terminal domain, the structure of which has only been recently elucidated by cryoelectron microscopy (9). It consists of three functionally important domains, first a cysteine-rich domain, which prevents P2X7R desensitization, second a cytoplasmatic cap that interacts with the short N-terminus of the P2X7R and contributes to the ion channel, and third a so-called ballast region (9). Further, the cytoplasmic C terminal domain of the P2X7R contains several consensus sequences for the interaction with other proteins and numerous amino acid residues, which are putative sites for covalent protein modification (9–11).

In mononuclear phagocytes extracellular ATP originating from activated cells or spilled cytoplasm of damaged cells is a well-known danger signal. It triggers the ionotropic function of the P2X7R, which lowers the intracellular concentration of K^+^ ions (12). A reduced K^+^ concentration is a major trigger for the assembly of the NLRP3 (NACHT, LRR and PYD domains-containing protein 3) inflammasome. The NLRP3 inflammasome is a multiprotein complex, that specifically activates caspase-1 enabling the proteolytic maturation and secretion of the pro-inflammatory cytokines interleukin (IL)-1β and IL-18 (12–15). Inflammasome activation can result in pyroptosis, a form of cell death that further promotes inflammation (15). Both cytokines, IL-1β and IL-18, are center stage in host defense against infections, which pose a major threat for trauma patients. While the protective functions of IL-1β and IL-18 are vital, overshooting cytokine release can induce systemic inflammation causing barrier dysfunction, shock, sepsis, and, eventually, life-threatening multi-organ dysfunction. This is for instance a frequent complication in patients with multiple traumata or those, who underwent major surgery (16). Hence it is conceivable, that several mechanisms evolved that control the IL-1β release, which have been comprehensively reviewed before (12–15).

Most of the mechanisms controlling the release of inflammasome-dependent cytokines also inhibit ATP-independent pathways. Interestingly, we discovered several pathways by which unconventional nicotinic acetylcholine receptors (nAChRs) specifically inhibit the ionotropic function of monocytic P2X7Rs, without impairing ATP-independent pathways of inflammasome activation that are typical for pathogens (17–19). This is of high clinical relevance, because, at least in the acute phase of trauma and major surgery, host defense against infection is desirable, while trauma-induced sterile inflammation should be avoided. We demonstrated that activation of monocytic nAChRs composed of subunits α9, α7 and/or α10 by classical agonists such as acetylcholine or nicotine efficiently inhibit ATP-induced ion currents at monocytic P2X7Rs by a ion flux-independent mechanism (17, 18). Further non-canonical endogenous agonists of these nAChRs were identified, including phosphocholine (PC), phosphatidylcholines (20) and other compounds with a PC head-group (17–19, 21). In contrast to classical agonists, these non-canonical agonists do not induce ion currents at human nAChRs heterologously expressed in *Xenopus laevis* oocytes but seem to function as silent agonists or weak antagonists (18, 19). Interestingly, the acute-phase reactants C-reactive protein (CRP) (22), α1-antitrypsin (AAT) (23), and secretory leukocyte protease inhibitor (SLPI) (24) also activate monocytic nAChRs to control the function of the P2X7R (25). This suggests that these proteins are part of negative feed-back loops limiting systemic inflammation (25). None of these nAChR agonists induce ion currents at monocytic nAChRs, but metabotropically inhibit the ionotropic function of the P2X7R.

The purpose of this study is to elucidate key steps of the signal transduction mechanism that links activation of nAChRs to the inhibition of the ionotropic function of the P2X7R and, hence, to an inhibition of NLRP3 inflammasome activation and IL-1β release. We provide evidence that agonists of nAChRs activate the endothelial NO synthase (eNOS, *NOS3*), which results in a modification of C377 in the C terminal cytoplasmic domain of the P2X7R and, thus, in an inactivation of its ionotropic function.

## Materials and methods

### Chemicals and reagents

ACh chloride (Cat# A6625), apyrase (Cat# A6410), adenosine 5′-triphosphate (ATP) disodium salt hydrate (Cat# A2383), bovine serum albumin (BSA, Cat# A9418), choline chloride (Cat# C7017), CRP (Cat# AG723-M), dimethyl sulfoxide (DMSO, Cat# D2650), lipopolysaccharide (LPS, *E. coli* O111:B4, Cat# L2630; *E. coli* O26:B6, Cat# L2654), macrophage colony-stimulating factor (M-CSF, Cat# SRP3110), nicotine hydrogen tartrate salt (Cat# N5260), nigericin sodium salt (Cat# N7143), Nω-nitro-L-arginine methyl ester hydrochloride (L-NAME, Cat# N5751), phorbol 12-myristate 13-acetate (PMA, Cat# P1585), PC chloride calcium salt tetrahydrate (Cat# P0378), recombinant mouse interferon-γ (INF-γ, Cat# IF005), S-nitroso-N-acetyl-DL-penicillamine (SNAP, Cat# 3398), SIN-1 hydrochloride (Cat# 567028), and tetracycline hydrochloride (Cat# T7660) were purchased from Merck (Darmstadt, Germany). All chemicals for saline Ringer’s buffer preparations were purchased from Merck (Darmstadt, Germany). 1400W dihydrochloride (Cat# 1415), L-NIO dihydrochloride (Cat# 0546), and N^ω^-propyl-L-arginine hydrochloride (N-PLA, Cat# 1200) were purchased from Tocris (Bio-Techne, Wiesbaden-Nordenstadt, Germany). Gibco penicillin-streptomycin solution and L-glutamine solution were purchased from Thermo Fisher Scientific (Dreieich, Germany). BzATP (2’(3’)-O-(4-benzoyl-benzoyl)ATP trieethylammonium salt) was purchased from Jena Bioscience (Jena, Germany), 0.5 M ethylenediaminetetraacetic acid (EDTA) solution from bioWORLD (Dublin, OH, United States, Cat# 40520000), Hygromycin B Gold solution (Cat# ant-hg-1) and blasticidin solution (Cat# ant-bl-05) from InvivoGen (Toulouse, France), and recombinant human INF-γ from R&D Systems (Minneapolis, MN, United States; Cat# 285-IF-100). PMA and SNAP were dissolved in DMSO. Nigericin was dissolved in ethanol (EtOH). When appropriate, control experiments were performed with the corresponding concentrations of DMSO/EtOH without drugs.

### U937 cells

Monocytic U937 cells were obtained from the German Collection of Microorganisms and Cell Cultures (Braunschweig, Germany). U937 cells were cultured in Gibco™ RPMI 1640 medium (Thermo Fisher Scientific, Cat# 11530586) supplemented with 10% fetal calf serum (FCS; CellConcepts, Umkirch, Germany) under 5% CO_2_ atmosphere at 37°C as described before (18, 19). In some experiments the expression of eNOS (*NOS3*) in U937 cells was reduced by using siRNA technology. For this purpose, U937 cells were transfected with ON-TARGETplus human eNOS siRNA SMARTpool (Thermo Fisher Scientific). To test for unspecific effects of siRNA transfection, cells were transfected in parallel with negative control ON-TARGETplus Non-targeting Control Pool (Thermo Fisher Scientific). The transfection was performed in accordance with the manufacturer’s protocol and as described previously (18, 19). In brief, U937 cells were transfected with 30 pM siRNA/1 × 10^6^ cells using the Amaxa Cell Line Nucleofector Kit C and Nucleofector II Device (both from Lonza Cologne, Cologne, Germany). The transfected cells were then cultured for 48 h under 5% CO_2_ atmosphere at 37°C.

To investigate IL-1β release, untreated or transfected cells were resuspended on the day of the experiments in fresh RPMI medium + 10% FCS. 1 × 10^6^ cells/ml were transferred per well to 12-well plates (Greiner Bio-One, Frickenhausen, Germany) and stimulated with 1 µg/ml LPS (*E. coli* O26:B6) for 5 h under 5% CO_2_ atmosphere at 37°C. Thereafter, BzATP (100 µM) was added for 30 min in the presence or absence of cholinergic agonists, NOS inhibitors or NO donors. At the end of the experiments, cells were spun down (500 g, 8 min, 4°C) and the cell-free supernatants were collected and stored at −20°C for later cytokine and lactate dehydrogenase (LDH) measurements.

### THP-1 cells

THP-1 cells were obtained from the German Collection of Microorganisms and Cell Cultures and cultured under 5% CO_2_ atmosphere at 37°C using RPMI 1640 medium from Sigma (Merck, Cat# R8758) supplemented with 10% FCS from Capricorn (Ebsdorfergrund, Germany, Cat# FBS-16A). To investigate IL-1β release, monocytic THP-1 cells were resuspended on the day of the experiments in FCS-free RPMI medium, and 0.5 × 10^6^ cells/0.5 ml were transferred per well to 48-well plates (Greiner Bio-One). Thereafter, cells were treated with 1 µg/ml LPS (*E. coli* O26:B6) and cultured for 5 h under 5% CO_2_ atmosphere at 37°C as described previously (26). After priming, BzATP (100 µM) was added for 40 min in the presence or absence of cholinergic agonists, NOS inhibitors, or NO donors. At the end of the experiments, cells were spun down (500 g, 8 min, 4°C) and the cell-free supernatants were collected and stored at −20°C for later cytokine measurements.

Monocytic THP-1 cells acquire a macrophage-like phenotype by exposure to PMA (27). To obtain THP-1 cell-derived M1-like macrophages, a previously described protocol (26) was used with modifications. Monocytic THP-1 cells were resuspended in RPMI 1640 medium (Sigma) supplemented with 10% FCS (Capricorn), 50 U penicillin/ml and 50 µg streptomycin/ml. 0.3 × 10^6^ cells/ml and per well were seeded in 12-well plates (Greiner) and incubated with 50 nM PMA for 24 h, followed by 24 h incubation in fresh complete medium without PMA. Thereafter, to induce cell differentiation into M1-like macrophages, cells were cultured in fresh complete medium supplemented with 10 ng/ml human IFN-γ and 10 ng/ml LPS (*E. coli* O111:B4) for 48 h. To investigate IL-1β release on day 5, the medium was replaced by fresh RPMI 1640 medium (Sigma) supplemented with 10% FCS. The cells were stimulated with 1 µg/ml LPS (*E. coli* O26:B6) for 5 h and handled as described for monocytic THP-1 cells. The identity of M1-like macrophages on day 5 was evaluated by flow cytometry (see below). For this purpose, M0-like macrophages were differentiated in parallel to M1-like macrophages, using the above-described protocol without adding human IFN-γ and LPS. Cells were washed once with Dulbecco´s phosphate buffered saline without calcium and magnesium (PBS; Merck Cat# D8537) and detached using TrypLE™ Express (Thermo Fisher Scientific, Cat# 12605010) according to the manufacturer’s protocol. The cell number was determined, and cells were stored on ice until flow cytometry.

### Murine mononuclear leukocytes and bone marrow-derived macrophages

Male and female wild-type mice from The Jackson Laboratory (C57BL/6J), *Nos3* gene-deficient mice (C57BL/6.129/Ola-eNOS™) (28) and corresponding wild-type mice were used for isolation of peripheral blood mononuclear leukocytes (PBMCs). Experimental animals received humane care according to NIH “Guide for the Care and Use of Laboratory Animals”, and animal experiments were approved by the local authorities (University Düsseldorf, reference No. O16/04; Regierungspräsidium Giessen, Germany, reference no. 571_M). Mice were euthanized and blood was immediately drawn from the caval vein into heparinized syringes (2 ml syringes filled with 15 µl heparin solution (5.000 I.E/ml) from Ratiopharm, Ulm, Germany (Cat# 03029843). PBMCs were separated using discontinuous Percoll (Ge Healthcare Bio-Sciences AB, Uppsala, Sweden; 1.082 g/ml) density gradient centrifugation as described before (18). 0.1 x 10^6^ cells/0.1 ml were seeded in 96-well plates (Greiner) and cultured for 2 h in RPMI 1640 medium (Gibco or Sigma) at 5% CO_2_ at 37°C. Thereafter, non-adherent cells were removed, and fresh RPMI medium was added. For investigation of IL-1β release, BzATP (100 μM) or ATP (1 mM) was added for 30 min in the presence or absence cholinergic agonists or NO donors. Subsequently, cell culture supernatants were collected and stored at −20°C for later cytokine measurements.

Male and female C57BL/6J wild-type mice were used for bone marrow cell isolation and bone marrow-derived macrophage (BMDM) differentiation using a previously published protocol with modifications (26). 1 × 10^6^ bone marrow cells/ml complete medium [RPMI 1640 medium (Cytiva, Marlborough, MA, United States) supplemented with 10% FCS (CellConcepts), 50 U penicillin/ml, 50 µg streptomycin/ml and 10 ng/ml M-CSF were seeded in in Costar 12-well plates (Corning, NY, United States) and cultured for 72 h. Thereafter, to induce cell differentiation into M1-like macrophages, 1 ml of fresh complete medium was added supplemented with a final concentration of 10 ng/ml M-CSF, 10 ng/ml IFN-γ and 10 pg/ml LPS (*E. coli* O26:B6). Cells were cultured for another period of 72 h. On day 6 of cultivation, BMDMs were stimulated with 1 µg/ml LPS (*E. coli* O26:B6) for 5 h. Thereafter, ATP (1 mM) or nigericin (50 µM; together with 0.5 U/ml apyrase) was added in the absence or presence of the NO donors SNAP (10 mM) or SIN-1 (1 mM). Cell-free supernatant was harvested 40 min later and stored at −20°C for IL-1β measurements.

### Human primary monocytic cells

Blood samples were obtained from healthy (self-reported) female and male non-smoking adult volunteers. The study was performed in accordance with the Helsinki Declaration and approved by the ethics committee of the medical faculty Giessen, Germany (No. 90/18). Blood was drawn into sterile syringes containing 1 mM EDTA per ml blood. Monocytes were enriched from whole blood by negative selection using the RosetteSep™ human Monocyte Enrichment Cocktail and Lymphoprep™ density gradient (both from Stemcell Technologies, Cologne, Germany) according to the manufacturer’s protocol. Thereafter, enriched monocytes were stimulated with 5 ng/ml LPS (*E. coli* O26:B6) for 25 min during the first washing step. After centrifugation at 250 g (15 min, room temperature) and a second wash followed by centrifugation at 300 g (10 min, room temperature), the cells were resuspended in RPMI medium (Sigma). The purity of monocytes was evaluated by flow cytometry (see below).

To investigate IL-1β release, 1 × 10^5^ cells/250 µl RPMI medium were seeded in 96-well plates and cultured at 37°C, 5% CO_2_ for 3 h in LPS-free medium. Thereafter, the cells were stimulated with BzATP (100 µM) or nigericin (25 µM plus 0.5 U/ml apyrase) in the presence or absence SNAP or SIN-1. After another 30 min of incubation, cell-free cell culture supernatants were harvested and stored at −20°C until measurement of IL-1β concentrations and LDH activity.

### Measurement of IL-1β concentration and LDH activity

IL-1β concentrations in cell-free supernatants obtained in experiments on human monocytic U937 cells, THP-1 cells (monocytic, macrophage-like), and human primary monocytic cells were measured using the Human IL-1 beta/IL-1F2 DuoSet enzyme-linked immunosorbent assay (ELISA) from R&D Systems (Cat# DY201) according to the supplier’s instructions. In samples obtained in the mouse PBMC experiments, IL-1β concentrations were measured by using the Mouse Quantikine IL-1β Immunoassay (R&D Systems Cat# MLB00C), whereas samples obtained in the mouse BMDM experiments were measured by using the Mouse IL-1 beta/IL-1F2 DuoSet ELISA (R&D Systems Cat# DY401). To test for cell viability at the end of the cell culture experiments, the CytoTox 96^®^ Non-Radioactive Cytotoxicity Assay (Promega, Madison, WI, United States; Cat# G1780) was used to measure LDH activity according to the supplier’s instructions. LDH activities determined in cell-free supernatants are given as percentage of the total LDH activity of lysed control cells (**Supplementary Table S1**).

### RNA isolation, cDNA synthesis and real-time RT-PCR

To confirm efficient and selective silencing of human eNOS mRNA expression by siRNA, real-time RT-PCR was performed on siRNA transfected U937 cells and corresponding controls. First, total RNA was isolated from 1 × 10^6^ U937 cells using the RNesay Plus Mini Kit (Qiagen, Hilden, Germany) according to the manufacturer’s instructions. The obtained RNA (1 µg) was reversely transcribed using M-MLVH Reverse Transcriptase and 1 µg of random hexamer primers (Promega, Mannheim, Germany). Thereafter, real-time RT-PCR was performed (n = 4 per experimental group, each sample was assessed in duplicates) using the ABI 7900 Sequence Detection System (Applied Biosystems, Foster City, CA, USA) and Platinum SYBER green qPCR Super Mix-UDG (Invitrogen, Karlsruhe, Germany). Primers specific for human eNOS (fwd: 5´-AGC ACT GAG ATC GGC ACG AGG A-3´; rev: 5´-TGC TGC CTT GTC TTT CCA CAG GG-3´), human neuronal NOS (nNOS, *NOS1*; fwd: 5´-CCA CGG CCC ACG GGA TGT TC-3´; rev: 5´-CTC GGA AGT CGT GCT TGC CGT-3´), and the house keeping gene hydroxymethylbilane synthase (HMBS; fwd: 5´-CCC ACG CGA ATC ACT CTC AT-3´; rev: 5´-TGT CTG GTA ACG GCA ATG CG-3´) (17) were synthesized by Eurofins Genomics (Ebersberg, Germany). As a negative control, samples were run under the same conditions, using nuclease-free water instead of cDNA. Changes in the mRNA expression levels of eNOS were estimated by the 2^ΔCT^ method, where ΔCT represents the difference between the CT value of HMBS gene and the CT value of gene of interest (17, 19, 24). The mean of the mRNA expression values from cells transfected with non-targeting siRNA was set to one and the values from cells transfected with siRNA specific for eNOS were calculated accordingly.

### Flow cytometry

Flow cytometry analyses were performed to evaluate cell surface marker expression of THP-1 cell-derived macrophages (M0- and M1-like) and the purity of RosetteSep™ enriched human monocytes. THP-1 cell-derived macrophages were characterized by using a panel of antibodies against cluster of differentiation (CD)14, CD38, CD80 and HLA-DR (see **Supplementary Table S2**).

Human monocytes can be classified into three main subsets, classical (~85%), intermediate (~5%), and non-classical (~10%) monocytes, which are characterized by their level of CD14 and CD16 expression (29). A previously published gating strategy for whole blood flow cytometry to identify and characterize human monocyte subsets (30) was modified and used to analyze the purity of RosetteSep™ enriched human monocytes. To assess the monocyte subset proportions a panel of antibodies was used (see **Supplementary Table S2**). Antibodies against T cells (CD3) and B cells (CD19) were used to assess lymphocytic contaminations. The purity of the enriched cells was displayed by using a CD14/CD16/CD45/HLA-DR panel.

Cells were resuspended in flow cytometry buffer (PBS, 2 mM EDTA, 5% FCS). Thereafter, 5 × 10^5^ cells were incubated with appropriate antibodies for 30 min on ice. All antibodies (**Supplementary Table S2**) were used at the manufacturer’s recommended dilution or titrated to determine the optimal staining concentrations (between 1:20 and 1:100). Cells were also incubated with the control isotype antibody corresponding to each primary antibody. Thereafter, cells were washed twice in flow cytometry buffer. Then, the cells were fixed with 1% dissolved paraformaldehyde (PFA) before flow cytometry analysis.

Cells were acquired on a BD FACSVerse™ System (BD Biosciences) using the BD FACSuite™ software, recording at least 10,000 events for each sample. Forward scatter (FSC) files were exported and analyzed in FlowJo software version 10.8.1 (FlowJo, LLC). Monocytes and macrophages were gated by FSC–area (FSC-A) and side scatter–area (SSC-A), then single cells by SSC-A and side scatter-width (SSC-W). Surface marker-positive cells were gated based on Fluorescence Minus One (FMO) or isotype controls and the percentage of surface marker positive cells was measured and exported.

### Heterologous expression of human P2X7R and two-electrode voltage-clamp measurements

Electrophysiological two-electrode voltage-clamp (TEVC) measurements were performed on *Xenopus laevis* oocytes stages V and VI purchased from Ecocyte Bioscience (Castrop-Rauxel, Germany). Oocytes were injected with cRNA (0.35 ng per oocyte) encoding for the human P2X7R as previously described (31). In brief, the oocytes were placed in a perfusion chamber, perfused at room temperature with a Ca^2+^-free frog Ringer’s solution containing (mM): 100 NaCl, 2.5 KCl, 5 HEPES (4-(2-hydroxyethyl)-piperazine-1-ethanesulfonic acid) and 0.1 flufenamic acid (pH 7.4), and impaled with two 1 M KCl-filled electrodes. The membrane voltage was clamped to −40 mV and the transmembrane ion currents (I_M_) were recorded.

When the I_M_ was stabilized, BzATP (dissolved in frog Ringer’s solution to a working solution of 10 µM) was applied for 2 min (31). After a wash out period, the oocytes were transferred to a 24-well plate and incubated for 2 h at room temperature in either oocyte Ringer’s solution or Ringer’s solution supplemented with 1 mM SIN-1. Subsequently, oocytes were placed in the perfusion chamber, voltage clamped to −40 mV and the BzATP-induced changes in I_M_ were determined for a second time. Measurements were performed on oocytes from at least two different *Xenopus laevis* frogs.

### HEK293 cell lines stably expressing wild-type or mutated human P2X7R upon stimulation with tetracycline

The generation of a stable Tet-on HEK293 cell line expressing the full-length wild-type human P2X7R (hP2X7R) under the control of the tetracycline-inducible tetR promotor (wt-hP2X7-HEK) has been described previously (32). In the same wt-hP2X7R pcDNA5/FRT/TO plasmid used before, we mutated C377 or C388 singly to alanine using the QuikChange site-directed mutagenesis protocol (33) with Phusion high-fidelity DNA polymerase and Dpn I restriction endonuclease (both from New England BioLabs, Schwalbach, Germany). After verification of the DNA sequences by commercial nucleotide sequencing (Eurofins), we generated stable C377A (hP2X7R_C377A-HEK) and C388A-hP2X7R-HEK cell lines using the Flp-InTM T-RExTM-system (Invitrogen, Thermo Fisher Scientific).

### Intracellular Ca^2+^ imaging

Changes in intracellular Ca^2+^ concentrations ([Ca^2+^]_i_) were imaged in wild-type human P2X7R-HEK cells as previously described (32). Cells were seeded in high glucose DMEM (Gibco) supplemented with 10% FCS (Biochrome) and 2 mM L-glutamine (Gibco) in glass bottom culture dishes (CELLview™, Greiner Bio-One, Kremsmünster, Austria) and grown for 24 – 48 h. To induce P2X7R expression, 1 µg/ml tetracycline was added, and cells were cultured for another 24 – 48 h at 37°C, 5% CO_2_. To image [Ca^2+^]_i_, the culture medium was replaced by a saline bath Ringer’s solution containing (in mM): 5.4 KCl, 120 NaCl, 2 CaCl_2_, 1 MgCl_2_, 10 HEPES, 25 glucose (pH 7.4). Imaging was performed at room temperature and as described previously (22, 32). Three independent batches of P2X7R-HEK cells were used in these experiments and a total number of 411 cells were tracked individually.

### Whole-cell patch-clamp measurements on human wild-type P2X7R and P2X7R mutants

Whole-cell patch-clamp measurements were performed on wt-hP2X7R-HEK, hP2X7R_C377A-HEK and hP2X7R_C388A-HEK cells. The cells were seeded in high glucose DMEM (Gibco) supplemented with 10% FCS (Biochrome) and 2 mM L-glutamine (Gibco) in cell culture dishes (Nunc, Roskilde, Denmark) and cultured at 37°C, 5% CO_2_ for 24 – 48 h. P2X7R expression was induced by adding 1 µg/ml tetracycline (Sigma-Aldrich) and a subsequent incubation for 24 – 48 h, as described previously (32). For the measurements, DMEM was replaced by bath solution (for details see [Ca^2+^]_i_ measurements) and whole-cell patch clamp recordings were performed as described previously (22, 31, 32). The whole-cell recordings were performed at room temperature. The membrane potential was clamped to −60 mV and the whole-cell transmembrane currents (measurement I) were recorded. BzATP (100 µM) and SIN-1 (1 mM) were dissolved in bath solution and applied by using a pressure-driven microperfusion system (measurement II). At least three independent batches of wt-hP2X7R-HEK, hP2X7R_C377A-HEK and hP2X7R_C388A-HEK cells were used in these experiments.

### Structural protein images of the rat P2X7R

Structural images of the rP2X7R protein were generated with the molecular visualization system PyMOL by Schrödinger (2022, Version 2.5) using the cryo-EM PDB files PDB 6u9v and 6u9w for the closed and open structures of rP2X7R, respectively (9).

### Statistical analyses and data processing

Results obtained in IL-1β release or electrophysiological experiments were analyzed using SPSS^®^ (Version 23, IBM^®^, Armonk, NY, USA). Multiple independent data sets were first analyzed by the non-parametric Kruskal–Wallis test. In case of a p ≤ 0.05, the non-parametric Mann–Whitney U test was performed to compare between individual groups and again, a p ≤ 0.05 was considered as evidence for statistical significance. Dependent data sets were analyzed first by the Friedman test followed by the Wilcoxon signed-rank test. All numbers (n) of the individual experiments are indicated in the Results section and in the Figures. The n-number given in experiments on primary cells represent data obtained from the cells of individual mice or humans. In experiments with cell lines, the n-number refers to independent experiments performed on different days and with different cell passages. No outliers were excluded from the analyses. An exception of this principle are cells used for electrophysiological measurements that lost voltage-clamp or membrane seal, or otherwise failed to remain viable throughout the measurements, and thus were excluded from the analyses. The free and open-source software Inkscape version 0.48.5 r10040 (licensed under the GPL) was used for visualization of the data.

## Results

**Figure 1:**
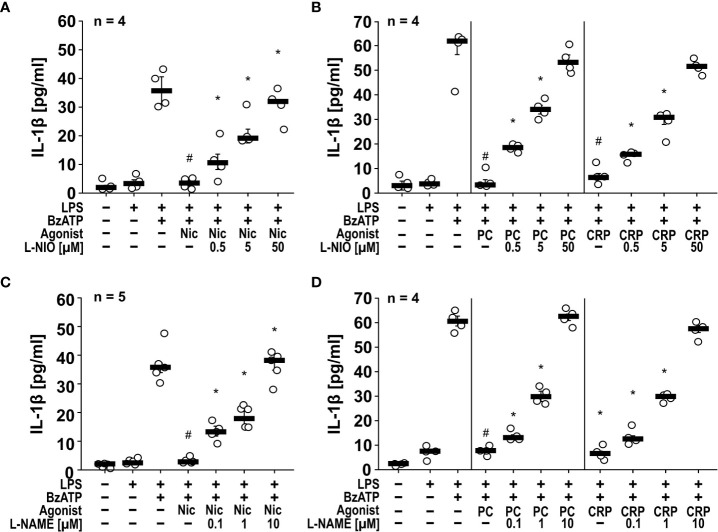
The inhibitory potential of nicotine (Nic), phosphocholine (PC) and C-reactive protein (CRP) on the BzATP-induced release of IL-1β by human monocytic U937 cells is prevented by nitric oxide synthase (NOS) inhibitors. U937 cells were primed with LPS (1 µg/ml) for 5 h, and BzATP (2’/3’-O-(4-benzoylbenzoyl)adenosine-5’-triphosphate, tri(triethylammonium) salt; 100 µM) was added for another 30 min to trigger IL-1β release, which was measured by ELISA. The inhibitory potential of the nicotinic acetylcholine receptor (nAChR) agonists Nic (100 µM), PC (100 µM) and CRP (5 µg/ml) on the BzATP-induced release of IL-1β was investigated in absence and presence of the NOS inhibitors **(A, B)** L-NIO (N5-(1-iminoethyl)-L-ornithine dihydrochloride) and **(C, D)** L-NAME (N-omega-nitro-L-arginine methyl ester hydrochloride). The inhibitory potential of nAChR agonists on the BzATP-induced release was concentration-dependently prevented by all NOS inhibitors. **(A, C)** Some IL-1β values (untreated, LPS, LPS+BzATP, LPS+BzATP+Nic) served as controls for multiple NOS inhibitors (**Supplementary Figures S1A, C**). Data are presented as individual data points, bars represent median, whiskers percentiles 25 and 75. # p ≤ 0.05 significantly different from samples, in which BzATP was given alone, *p ≤ 0.05 significantly different from BzATP + nicotine. Kruskal-Wallis followed by Mann-Whitney rank sum test.

*Inhibition of NOS reverts the inhibitory effects of nAChR agonists on IL-1β release.* To test the hypothesis, that NOS are involved in the inhibitory effect of nAChR agonists on the ATP-induced release of IL-1β by monocytic cells, we made use of the NOS inhibitors L-NIO (34) (**Figures 1A, B**), L-NAME (35) (**Figures 1C, D**), N-PLA (**Supplementary Figures S1A, B**) and 1400 W (**Supplementary Figures S1C, D**). Cells of the human monocytic U937 cell line were primed with LPS (1 µg/ml) for 5 h to induce the expression of pro-IL-1β and of essential compounds of the NLRP3 inflammasome, followed by a second stimulation with the P2X7R agonist BzATP (100 µM) for 30 min to induce inflammasome assembly. These experiments were performed in the presence or absence of nicotine (100 µM), PC (100 µM) or CRP (5 µg/ml). Thereafter, IL-1β was measured in the cell-free culture medium. As described before (17, 18, 22), supernatants of unstimulated cells and cells primed with LPS contained only trace-amounts of IL-1β, while supernatants of cells treated with both LPS and BzATP released 30 – 70 pg/ml IL-1β. As expected (17, 22), the BzATP-induced release of IL-1β was significantly (p ≤ 0.05) inhibited by nicotine (n = 5), PC (n = 4) and CRP (n = 4) (**Figure 1**, **Supplementary Figure S1**). In line with our hypothesis, all investigated NOS inhibitors dose-dependently prevented the effects of the nicotinic agonists (**Figure 1**, **Supplementary Figure S1**). In the absence of nicotine, PC or CRP, NOS inhibitors did not affect the BzATP-induced release of IL-1β (n = 4, not shown).

**Figure 2:**
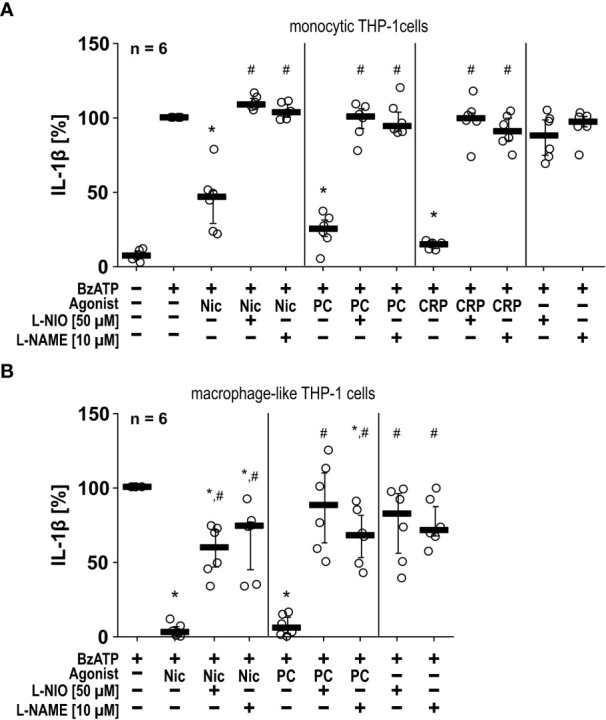
The inhibitory potential of nicotine (Nic), phosphocholine (PC) and C-reactive protein (CRP) on the BzATP-induced release of IL-1β by human monocytic and macrophage-like THP-1 cells is reversed by nitric oxide synthase (NOS) inhibitors. Monocytic **(A)** and differentiated macrophage-like **(B)** THP-1 cells were used. Cells were primed with lipopolysaccharide (LPS; 1 µg/ml, 5 h). Thereafter, the P2X7 receptor agonist BzATP ((2’/3’-O-(4-benzoylbenzoyl)adenosine-5’-triphosphate, tri(triethylammonium) salt) was added for another 40 min to trigger IL-1β release, which was measured by ELISA. The inhibitory potential of Nic (100 µM), PC (200 µM) and CRP (10 µg/ml) on the BzATP-induced release of IL-1β was investigated in absence and presence of the NOS inhibitors L-NIO (50 µM; N5-(1-iminoethyl)-L-ornithine dihydrochloride) or L-NAME (10 µM; N-omega-nitro-L-arginine methyl ester hydrochloride). **(B)** In experiments on macrophage-like THP-1 cells, the concentration of IL-1β released in response to BzATP was calculated by subtracting the IL-1β concentrations measured in supernatants of cells treated with LPS alone. In each experiment, the IL-1β concentration obtained after stimulation with BzATP was set to 100% and all other values were calculated accordingly. Data are presented as individual data points, bars represent median, whiskers encompass the 25th to 75th percentile. *p ≤ 0.05, different from LPS-primed cells stimulated with BzATP alone; #p ≤ 0.05 significantly different from samples, in which BzATP plus agonist was given. Friedman test followed by the Wilcoxon signed-rank test.

To test if NOS inhibitors also function in another independent monocytic cell line, we repeated some of the experiments with human monocytic THP-1 cells and differentiated macrophage-like THP-1 cells (**Figures 2A, B**). Like U937 cells, monocytic and macrophage-like THP-1 cells primed with LPS and stimulated with BzATP released IL-1β (monocytic = 109 ± 45 pg/ml, n = 6; macrophage-like = 512 ± 217 pg/ml, n = 6), which was significantly inhibited by nicotine, PC and CRP (**Figures 2A, B**). In THP-1 cells, the NOS inhibitors L-NIO (50 µM) and L-NAME (10 µM) also significantly reverted the effects of all nAChR agonists tested (**Figures 2A, B**). Flow cytometrical analyses confirmed that differentiated THP-1 cells expressed cell surface markers typical for M1-like macrophages (**Supplementary Figure S2**).

**Figure 3:**
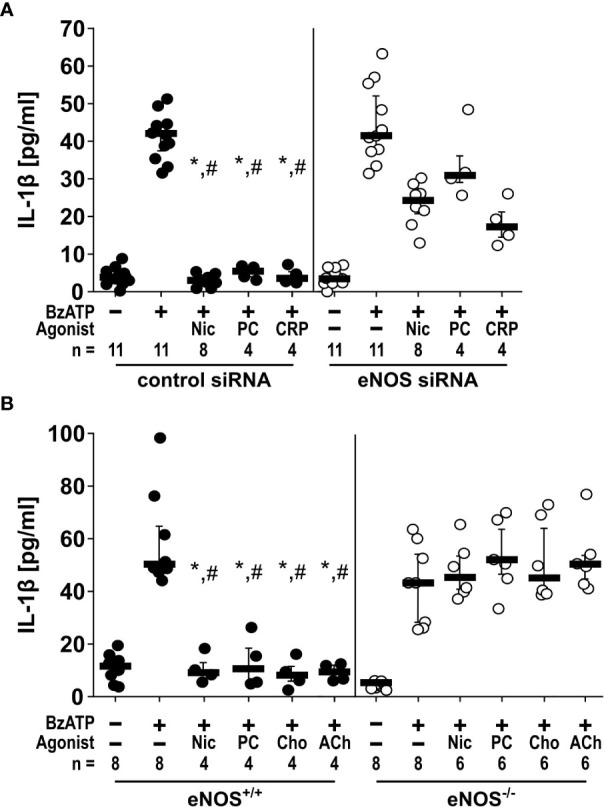
The inhibitory potential of cholinergic agonists on BzATP-induced IL-1β release by monocytic cells depends on endothelial nitric oxide synthase (eNOS, *NOS3*) activity. **(A)** U937 cells were transfected with siRNA targeting eNOS or with control siRNA. Forty-eight hours after transfection, cells were primed with lipopolysaccharide (LPS; 1 µg/ml) for 5 h and BzATP ((2’/3’-O-(4-benzoylbenzoyl)adenosine-5’-triphosphate, tri(triethylammonium) salt; 100 µM) was given for additional 30 min in the presence or absence of nicotinic agonists nicotine (Nic; 100 µM), phosphocholine (PC; 100 µM) or C-reactive protein (CRP; 5 µg/ml). While in cells transfected with control siRNA, the BzATP-induced release of IL-1 β was inhibited by nicotinic agonists, the inhibitory activity of all agonists was blunted in eNOS siRNA-transfected cells. Data were analyzed by Kruskal-Wallis test followed by Mann-Whitney rank sum test. **(B)** Freshly isolated mouse peripheral blood mononuclear cells (PBMCs) isolated from wild-type (eNOS^+/+^) mice and mice deficient in eNOS (eNOS^-/-^) were left untreated or stimulated with BzATP for 30 min, in the presence or absence of nicotinic agonists Nic, PC, choline (Cho; 100 µM) or acetylcholine (ACh; 10 µM). BzATP induced the release of IL-1β in PBMCs obtained from all mouse strains. Nicotinic agonists significantly inhibited the IL-1β release by PBMCs from eNOS^+/+^ mice. In contrast, the IL-1β release by PBMCs from eNOS^-/-^ mice was not affected by nicotinic agonists. Friedman test followed by the Wilcoxon signed-rank test. Data are presented as individual data points, bars represent median, whiskers percentiles 25 and 75. ∗p ≤ 0.05 significantly different from samples, in which BzATP was given alone #p ≤ 0.05 significantly different from corresponding samples control siRNA vs. eNOS siRNA and eNOS^+/+^ vs. eNOS^-/-^.

*The inhibitory effects of nAChR agonists on BzATP-induced IL-1β release depend on eNOS*. As NOS function seems to be very quickly activated in response to nAChR agonists, we did not investigate the involvement of iNOS which is constitutively active and regulated on the mRNA level. Therefore, we further investigated if eNOS and nNOS, the functions of which are regulated on the protein level (36, 37), might be involved in the control of BzATP-mediated IL-1β release. In real-time RT-PCR experiments on U937 cells primed with LPS, *NOS3* mRNA was detectable albeit at low levels, while *NOS1* mRNA was in the range of the threshold of detection. Therefore, we hypothesized, that the eNOS is an essential part of the signaling cascade linking nAChR activation to the inhibition of the ionotropic P2X7R function. To test this hypothesis, we followed two independent approaches. First, we transfected U937 cells with control siRNA or siRNA specific for the *NOS3* gene, which significantly (n = 8, p ≤ 0.05) down-regulated the mRNA expression of *NOS3* (**Supplementary Figure S3**). With these cells we performed the above-described IL-1β release experiments in the presence or absence of nAChR agonists. While transfection of control siRNA did not impair the inhibitory effect of nicotine, PC, and CRP on the BzATP-mediated release of IL-1β, transfection of siRNA specific for *NOS3* blunted the inhibitory effect (**Figure 3A**).

In a second approach, freshly isolated PBMCs from mice deficient in *NOS3* (C57BL/6.129/Ola-eNOS™) (28) and corresponding wild-type mice were used and left untreated or stimulated with BzATP for 30 min, in the presence or absence of nicotine, PC, choline (Cho; 100 µM), or acetylcholine (ACh; 10 µM). As expected, BzATP induced the release of IL-1β by PBMCs obtained from both mouse strains. Nicotinic agonists signifilicantly inhibited the BzATP-induced IL-1β release by PBMCs from wild-type mice. In contrast, the IL-1β release by PBMCs from *Nos3*-deficient mice was unaffected by all nicotinic agonists tested **(** **Figure 3B**).

**Figure 4:**
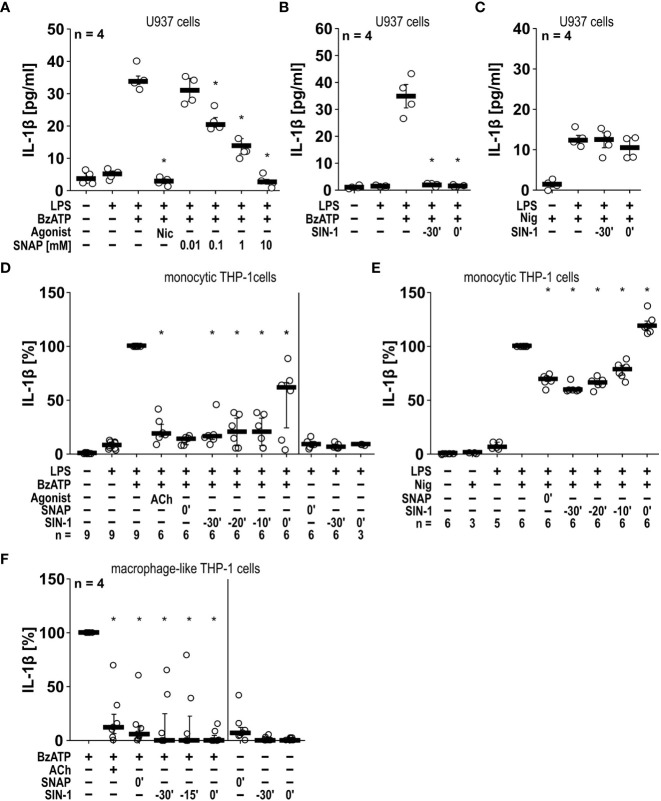
The BzATP-induced release of IL-1β by monocytic and macrophage-like cells is inhibited by the NO donors SNAP and SIN-1. **(A, B)** Monocytic U937 cells were primed with lipopolysaccharide (LPS; 1 µg/ml) for 5 h, and BzATP ((2’/3’-O-(4-benzoylbenzoyl)adenosine-5’-triphosphate, tri(triethylammonium) salt; 100 µM) was added for another 30 min to trigger IL-1β release, which was measured by ELISA. The NO donor SNAP (S-nitroso-N-acetyl-DL-penicillamine) concentration-dependently inhibited the BzATP-induced release of IL-1β like nicotine (Nic; 100 µM), which served as a positive control **(A)**. Addition of SIN-1 (1 mM) 30 min before BzATP (t = -30’) or shortly before BzATP (t = 0’) inhibited the BzATP-induced release of IL-1β **(B)**. U937 cells were primed with LPS for 5 h, and nigericin (Nig) was added together with apyrase (0.5 U/ml) for another 30 min to trigger IL-1 β release. The Nig-induced IL-1β release was unimpaired by SIN-1 **(C)**. Kruskal-Wallis followed by Mann-Whitney rank sum test **(A–C)**. **(D, E)** Similar results were found on LPS-primed monocytic THP-1 cells stimulated with BzATP **(D)** or Nig **(E)** for 40 min in the presence and absence of SNAP and SIN-1. Acetylcholine (ACh; 10 µM) was used as a positive control. The IL-1β concentration in supernatants of primed THP-1 cells stimulated with BzATP or Nig alone was set to 100%, and all other values were calculated accordingly. Friedman test followed by the Wilcoxon signed-rank test. **(F)** Macrophage-like THP-1 cells were left untreated or primed with LPS (1 µg/ml, 5 h). Thereafter, BzATP (100 µM) was added for another 40 min to trigger IL-1β release. The amount of IL-1β released in response to BzATP was calculated by subtracting the IL-1β concentrations measured in supernatants of cells treated with LPS alone. In the presence of ACh (10 µM) the BzATP-induced release of IL-1β was blunted. Application of the NO donors SNAP or SIN-1 at different time points, reversed the BzATP-induced release of IL-1β. Friedman test followed by the Wilcoxon signed-rank test. **(A–F)** Data are presented as individual data points, bars represent median, whiskers percentiles 25 and 75. ∗ p ≤ 0.05 significantly different from samples, in which BzATP or Nig was given alone.

*NO and peroxynitrite mimic the inhibitory effects of nAChR agonists on IL-1β release.* NOS catalyzes the conversion of L-arginine into NO and L-citrulline (34). Highly reactive peroxynitrite can be formed from NO in the presence of superoxide anions (38). To test our hypothesis that NO can inhibit the BzATP-induced release of IL-1β by LPS-primed monocytic U937 cells and THP-1 cells, we used the NO/peroxynitrite donors SNAP and SIN-1 (39) and performed IL-1β release assays in U937 cells (**Figures 4A-C**). In line with our hypothesis SNAP showed a dose-dependent inhibitory effect with first statistically significant effects at a 100 µM concentration (n = 4, p ≤ 0.05) and a virtually complete inhibition at 10 mM (n = 4, p ≤ 0.05; **Figure 4A**). In the same line, SIN-1 (1 mM) fully inhibited the BzATP-induced release of IL-1β, both when given together with BzATP or when applied 30 min earlier (n = 4, p ≤ 0.05, each; **Figure 4B**). When the IL-1β secretion was triggered by the pore-forming bacterial toxin nigericin (50 µM), SIN-1 had no effect on the release of IL-1β (**Figure 4C**). In these experiments, the ATP-degrading enzyme apyrase (0.5 U/ml), was added together with nigericin to ensure, that only ATP-independent mechanisms are investigated.

In the monocytic cell line THP-1 similar results were obtained (**Figures 4D, E**). SNAP (10 mM) and SIN-1 (1 mM) significantly inhibited the BzATP-mediated release of IL-1β by LPS-primed cells (n = 6, p ≤ 0.05, each). However, when SIN-1 was given together with BzATP, IL-1β levels showed a broader distribution, while 30 min preincubation with SIN-1 seemed to result in a more robust inhibitory effect. This prompted us to preincubate the monocytic THP-1 cells with SIN-1 20 and 10 min before application of BzATP, with similar results like those obtained after 30 min of preincubation (**Figure 4D**). When LPS-primed monocytic THP-1 cells were treated with SNAP or SIN-1 in the absence of BzATP, essentially no IL-1β was released. In contrast to U937 cells, application of SNAP significantly reduced the nigericin-induced release of IL-1β by LPS-primed THP-1 cells by about 40% (n = 6, p ≤ 0.05; **Figure 4E**). The same holds true for SIN-1, when preincubated for 30, 20 or 10 min before application of nigericin (n = 6, p ≤ 0.05, each; **Figure 4E**). When SIN-1 was, however, applied together with nigericin, the IL-1β levels in cell culture supernatants were even slightly but significantly increased (n = 6, p ≤ 0.05; **Figure 4E**). We also included experiments on THP1-derived M1-like macrophages, in which treatment with SNAP and SIN also significantly reduced the BzATP-induced release of IL-1β (n = 7, p ≤ 0.05; **Figure 4F**).

**Figure 5:**
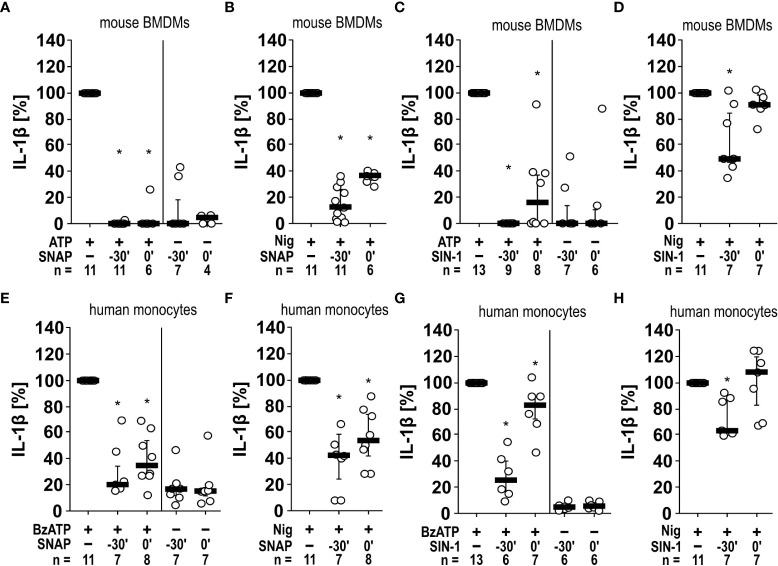
Inhibition of IL-1β release by the NO donors SNAP and SIN-1 in murine bone marrow-derived macrophages (BMDMs) and primary human monocytes. **(A–D)** Mouse BMDMs were primed with lipopolysaccharide (LPS; 1 µg/ml, 5 h). **(A, C)** Thereafter, ATP (1 mM) was added for another 40 min to trigger IL-1β release, which was measured by ELISA. Application of the NO donor SNAP (S-nitroso-N-acetyl-DL-penicillamine; 1 mM) 30 min prior to ATP (t = -30´) or shortly before ATP (t = 0´) reversed the ATP-induced release of IL-1β. Similar results were found by using SIN-1 (1 mM). **(B, D)** To test for P2X7 receptor-independent IL-1β release, nigericin (Nig; 50 µM) was added together with apyrase (0.5 U/ml) for 40 min to LPS-primed mouse BMDMs. The Nig-induced IL-1β release was unimpaired by SIN-1 at t = 0´ and slightly reversed in presence of SNAP and SIN-1 at t = -30´. The amount of IL-1β released in response to ATP/Nig was calculated by subtracting the IL-1β concentrations measured in supernatants of cells treated with LPS alone. ∗ p ≤ 0.05 significantly different from samples, in which ATP or Nig was given alone. **(E–H)** Similar experiments were performed on primary human monocytes enriched from freshly collected human whole blood. Cells were primed with LPS (5 ng/ml, 20 min pulse) during the enrichment process. After 3 h, BzATP ((2’/3’-O-(4-benzoylbenzoyl)adenosine-5’-triphosphate, tri(triethylammonium) salt; 100 µM); **(E, G)** or Nig **(F, H)** was added for another 30 min to trigger IL-1β release. The IL-1β concentration in experiments, in which primed cells were stimulated with BzATP or Nig alone, was set to 100% and all other values were calculated accordingly. ∗ p ≤ 0.05 significantly different from samples, in which BzATP was given alone. **(A–H)** Data are presented as individual data points, bars represent median, whiskers percentiles 25 and 75. Friedman test followed by the Wilcoxon signed-rank test.

Next, we tested, if SNAP and SIN-1 exerts the same effects on primary murine cells. Murine PBMCs freshly isolated from wild-type C57BL/6J mice were pulsed with LPS (10 ng/ml) before separation and cultured for 2 h. Non-adherent cells were removed, and remaining cells were stimulated with ATP (1 mM) for another 30 min. The IL-1β levels in cell culture supernatants were significantly (n ≥ 6, p ≤ 0.05, each) lower, when cells were pretreated with SIN-1 for 30 min before applying ATP or, when SIN-1 was given together with ATP (**Supplementary Figure S4**). In the absence of ATP, only very low amounts of IL-1β were detected, irrespective of the absence or presence of SIN-1 (**Supplementary Figure S4**). A more extended set of experiments was performed with murine BMDMs (**Figures 5A–D**). As shown before for U937 cells and THP-1 cells, SNAP efficiently and significantly (n ≥ 6, p ≤ 0.05) inhibited the ATP-induced release of IL-1β, when applied 30 min before or together with ATP but did not induce IL-1β release in the absence of ATP (**Figure 5A**). The same holds true for SIN-1 (n ≥ 6, p ≤ 0.05; **Figure 5B**). When SNAP was combined with nigericin and apyrase to investigate ATP-independent mechanisms of inflammasome activation and IL-1β secretion, the IL-1β release by murine BMDMs was also reduced but not fully abolished (n ≥ 6, p ≤ 0.05; **Figure 5C**). Incubation with SIN-1 for 30 min before application of ATP also reduced IL-1β levels (n ≥ 7, p ≤ 0.05), while SIN-1 application concomitantly with ATP did not (**Figure 5D**).

Finally, we enriched human monocytes from the blood of healthy volunteers using the RosetteSep™ technique to a purity of 76.07%, 77.33% and 79.51% as analyzed in samples from three volunteers by flow cytometry. Among these monocytes, 88.90 ± 2.20% (median ± standard error of mean) were classical, 4.20 ± 0.47% were intermediate, and 6.19 ± 2.15% were nonclassical monocytes (the gating strategy can be found in **Supplementary Figure S5**). Similar experiments like those performed on murine BMDMs were also performed with these cells using BzATP and nigericin plus apyrase (**Figures 5E–H**). Although the variability of the data was higher, essentially the same results were obtained for human monocytes as for murine BMBMs (**Figure 5**).

Taken together, SNAP and SIN-1 efficiently and consistently inhibit the ATP/BzATP-mediated release of IL-1β by monocytic cell lines, THP-1-dependent macrophages as well as by human and murine primary monocytes and macrophages. ATP-independent IL-1β release can also be reduced, however, this reduction is less prominent.

**Figure 6:**
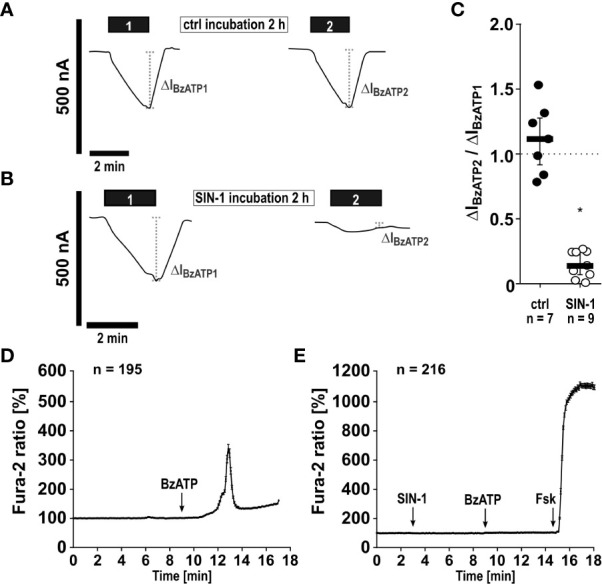
The NO and peroxynitrite donor SIN-1 inhibits BzATP-induced ion channel functions of heterologously expressed human P2X7 receptors (hP2X7R). **(A–C)** Two-electrode voltage-clamp measurements were performed on *Xenopus laevis* oocytes expressing the hP2X7R. **(A)** In control experiments (ctrl) application of the P2X7R agonist BzATP ((2’/3’-O-(4-benzoylbenzoyl)adenosine-5’-triphosphate, tri(triethylammonium) salt; 10 µM; 2 min; black bar “1”) resulted in a current stimulation (ΔI_BzATP1_). After a wash out period, the oocytes were incubated for 2 h in oocyte Ringer’s solution as a control (ctrl). Thereafter, the BzATP-induced effect was determined for a second time (black bar “2”; ΔI_BzATP2_). **(B)** After determination of the ΔI_BzATP1_, oocytes were incubated for 2 h with SIN-1 (1 mM). Subsequently, the ΔI_BzATP2_ was determined. **(C)** The normalized ΔI_BzATP_ values from experiments as shown in panel A and B (ΔI_BzATP2_/ΔI_BzATP1_) were statistically analyzed (∗ p ≤ 0.05 significantly different from ctrl). Data are presented as individual data points, bars represent median, whiskers encompass the 25^th^ to 75^th^ percentile. **(D, E)** Calcium imaging experiments were performed on hP2X7 receptor expressing HEK cells. Intracellular Ca^2+^ levels ([Ca^2+^]_i_) of HEK-P2X7R cells were recorded as Fura-2/AM (Fura-2) fluorescence intensity ratio of 340:380 nm excitation (mean ± SEM). **(D)** In control experiments (no SIN-1), application of BzATP (100 µM) induced a rise in [Ca^2+^]_i_. **(E)** Application of SIN-1 (1 mM) did not cause significant alterations in [Ca^2+^]_i_. In the presence of SIN-1 the BzATP-induced rise in [Ca^2+^]_i_ was blunted (p ≤ 0.05 significantly different from BzATP-induced effect in **(D)**. At the end of the experiment, a positive control for cell viability and the Ca^2+^ imaging setup was included: forskolin (Fsk, 40 µM) was applied to induce a cyclic adenosine monophosphate-triggered rise in [Ca^2+^]_i_. Friedman test followed by the Wilcoxon signed-rank test.

*SIN-1 inhibits the ionotropic function of P2X7Rs.* As NO and peroxynitrite cause protein nitration or nitrosylation and thereby modulate their function (39–44), we hypothesized that modification of the P2X7R inhibits its ionotropic function. To address this question, the human P2X7R was heterologously expressed in *Xenopus laevis* oocytes and TEVC measurements were performed. BzATP (10 µM) elicited non-desensitizing inward currents, which were repeatable after 2 h of incubation in ATP-free control solution (**Figures 6A, C**). The BzATP-induced ion current changes were drastically reduced, when oocytes were incubated with SIN-1 (1 mM) for 2 h in the same solution (n ≥ 7; p ≤ 0.001; **Figures 6B, C**).

**Figure 7:**
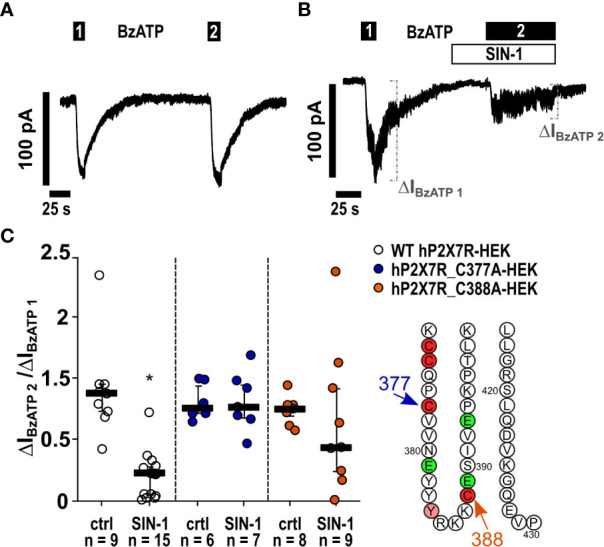
The NO and peroxynitrite donor SIN-1 inhibits the BzATP-induced ion channel functions in human P2X7 receptor (hP2X7R) expressing HEK cells *via* the cysteine C377 in the C-terminal intracellular loop. **(A, B)** Depicted are representative current curves of whole-cell patch-clamp measurements. **(A, C)** In control experiments (ctrl) on human wild-type (WT) P2X7R expressing HEK cells (WT hP2X7-HEK), consecutive application of the P2X7R agonist BzATP (100 µM) induced repetitive ion current stimulations (BzATP1 and 2). **(B, C)** In the presence of SIN-1 (1 mM), the BzATP-induced ion current was blunted. **(C)** Similar experiments were performed on HEK cells expressing hP2X7R mutants generated by replacing cysteine 377 or 388 by an alanine (hP2X7_C377A; hP2X7_C388A) in the C-terminal intracellular loop. The BzATP-induced current changes (ΔI_BzATP1_, ΔI_BzATP2_) were normalized (ΔI_BzATP2_/ΔI_BzATP1_). **(C)** All BzATP-induced current changes (ΔI_BzATP_) are shown as individual data points, bars represent median, whiskers encompass the 25th to 75th percentile. *p ≤ 0.05, Friedman test followed by the Wilcoxon signed-rank test.

In a similar experiment, changes in intracellular Ca^2+^ levels, which are caused by the BzATP-induced ion currents, were measured. In HEK cells stably expressing the tetracycline-inducible wild-type hP2X7R, Ca^2+^ transients were robustly induced by BzATP (**Figure 6D**). After preincubation with SIN-1, the BzATP-induced Ca^2+^ signals were virtually absent (**Figure 6E**) and significantly different from control experiments (**Figure 6D**; ∗ p ≤ 0.05). To study, if the cells are still able of eliciting Ca^2+^ transients after treatment with SIN-1, forskolin (40 µM) was added, which induced robust signals (**Figure 6E**). Finally, whole cell patch-clamp experiments were performed on HEK cells expressing the human wild-type *P2RX7* to investigate ion current changes in response to BzATP. As expected (32), BzATP (100 µM) induced repeatable inward currents (**Figures 7A, C**), which were significantly blunted, when SIN-1 (1 mM) was applied shortly before a second application of BzATP (n ≥ 9, p ≤ 0.05; **Figures 7B, C**).

**Figure 8:**
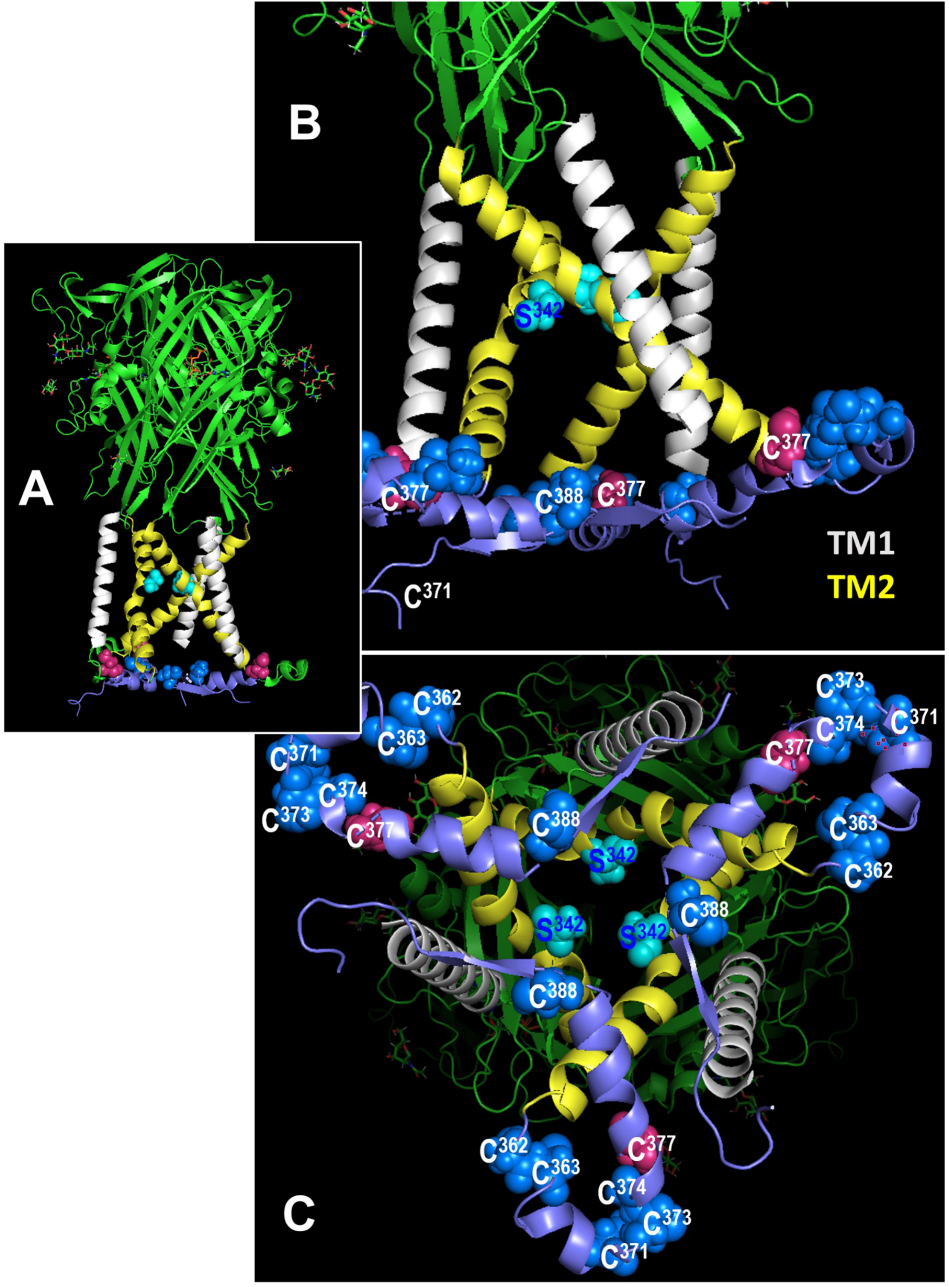
Location of cysteines C377 and C388 in the rat P2X7 receptor (rP2X7R) in the open channel conformation. In all figures, the C-terminal residues 400-595 to the end of the 595 residues long subunits are hidden for simplicity. **(A)** Side view of the large extracellular domain harboring the ATP binding sites, the six transmembrane domains (3 x TM1 in white, 3 x TM2, in yellow), and an interfacial helically structured domain lying parallel to the membrane plane. Also shown are the three S342 residues, which constitute the channel gate (46). **(B)** Enlarged side view of the transmembrane domains, the S342 residues (cyan) and the position of two (of three each) residues C377 and C388 in the interfacial region. The other blue spheres indicate additional cysteines of the cysteine-rich domain. **(C)** Perpendicular view from the cytoplasmic side on the numerous cysteine residues of the interfacial region and the transmembrane domains above.

*Mutation of C377 to alanine abolishes P2X7R sensitivity to SIN-1*. The C terminal cytoplasmic domain of the P2X7R constitutes about 40% of the whole P2X7 protein (45) and is highly conserved between rat (rP2X7R) and hP2X7R (80.17% amino acid sequence identity) (**Supplementary Figure S6**). The C terminal domain contains several domains that potentially interact with other proteins and numerous amino acid residues, which are putative sites for covalent protein modification (9, 10, 45). We tested the two cysteines C377 and C388 as possible targets for P2X7R modification. C377 is the last cysteine of six cysteine residues that, starting at C362 (**Figure 8**), form the so-called cysteine-rich domain of the P2X7R (9, 47). C377 and C388 are located in the same plane near the cytoplasmic end of the transmembrane domains (**Figure 8**). C377 is located on the outer surface of the endodomain, C388 in its interior, ~23 Å vertically below the gate defined mainly by the ring of three S342 residues (46), one from each of the three TM2 helices. To test if the cysteine at position C377 of the P2X7R is involved in signaling, hP2X7R_C377A-HEK cells were used with the C377 mutated to an alanine (C377A), which cannot be modified by nitrosylation. When the above-described kind of patch-clamp experiments were performed with the hP2X7R_C377A-HEK cells, the ion current response towards BzATP remained unchanged, which is in contrast to the wild-type hP2X7R (**Figure 7C**). Of note, consistent with previous studies on HEK cells expressing different mutant P2X7Rs the mutated P2X7 receptors were still able to channel (48). In the presence of SIN-1, however, the BzATP-induced ion current changes did not differ from control-treated cells (**Figure 7C**), suggesting, that C377 plays an essential role in the SIN-1-mediated effects. Similarly, the response to BzATP was unchanged in C388A-hP2X7R-HEK cells compared to wild-type P2X7R (**Figure 7C**). The response to BzATP in the presence of SIN-1, however, was variable with the C388A mutant, ranging from no change in ion currents to a more than twofold increased response (**Figure 7C**).

## Discussion

**Figure 9:**
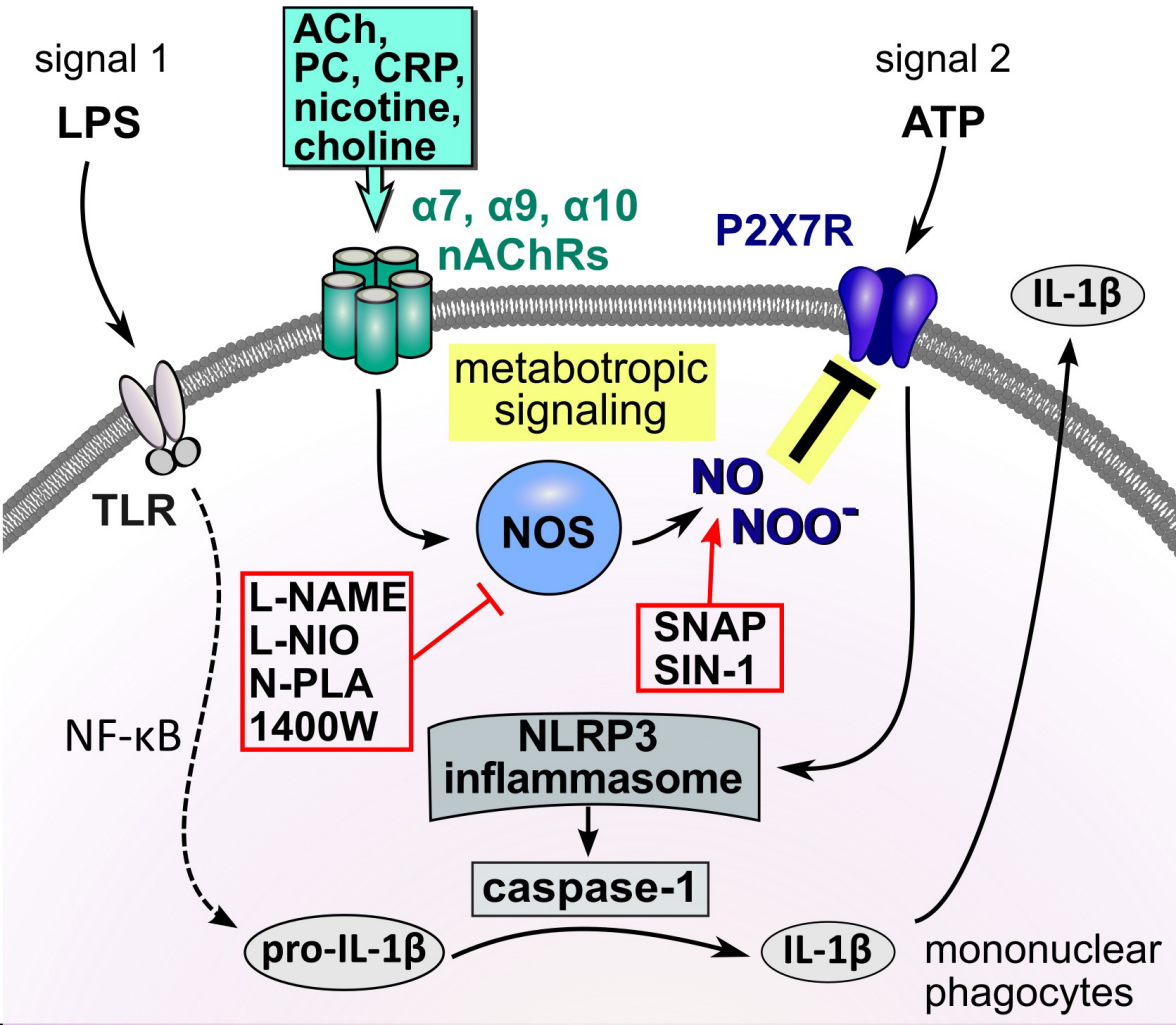
Schematic summary of the proposed metabotropic signaling mechanism. In mononuclear phagocytes extracellular ATP originating from activated cells or spilled cytoplasm of damaged cells trigger the ionotropic function of the P2X7R, resulting in NLRP3 inflammasome assembly, activation of caspase-1, cleavage of pro-IL-1β and release of bioactive IL-1β. Activation of nAChRs by classical and unconventional agonists down-regulates the response of the ATP-sensitive P2X7R, impairs NLRP3 inflammasome assembly and, consequently, the maturation as well as the release of IL-1β. We provide evidence that this inhibitory effect of nAChR stimulation on the secretion of IL-1β is mediated *via* endothelial NOS and modification of the P2X7R. ACh, acetylcholine; ATP, adenosine triphosphate; CRP, C-reactive protein; DAMP, danger-associated molecular pattern; IL-1β, interleukin-1β; LPS, lipopolysaccharide; nAChRs, nicotinic acetylcholine receptors; NF-κB, nuclear factor κB; NLRP3, NACHT, LRR, and PYD domains-containing protein 3; NOS, NO synthase; P2X7R, P2X7 receptor; PAMP, pathogen-associated molecular pattern; PC, phosphocholine; SNAP, S-nitroso-N-acetyl-DL-penicillamine; TLR, Toll-like receptors.

Activation of nAChRs in mononuclear phagocytes and epithelial cells down-regulates the response of the ATP-sensitive P2X7R, reduces NLRP3 inflammasome assembly and, consequently, the maturation as well as the release of IL-1β (17, 22, 23, 26). This mechanism is activated by several redundant endogenous signals (18, 19, 21–24, 26, 49) and, therefore, seems to be of high biomedical relevance. We demonstrated before, that signaling of AChRs in monocytes is ion flux-independent (17, 18, 22) and no changes in intracellular Ca^2+^ concentrations were detected (22). Flux-independent signaling mechanisms of nAChRs are poorly understood. In this study we investigated the mechanism of how activation of nAChRs translates into an inhibition of the ionotropic function of the P2X7R. It was shown before in neurons and endothelial cells, that activation of nAChR α7 can activate the function of NOS (37, 50–52) and that NO- or peroxynitrite-mediated modifications can regulate protein function (39, 43, 44, 53–57). Moreover, P2X7R was found to co-immunoprecipitate with nNOS in protein extracts of the prefrontal cortex (58). In endothelial cells, stimulation of nAChR α7 with nicotine results in the phosphorylation of eNOS at serine 1177 and induces the production of NO (59). These hints prompted us to hypothesize, that the inhibitory effect of nAChR stimulation on the secretion of IL-1β is mediated *via* NOS and NO- and/or peroxynitrite-mediated modification of the P2X7R (**Figure 9**).

In line with this hypothesis, we demonstrated that inhibitors of NOS efficiently and dose-dependently reversed the suppressive effect of nicotine, PC and native CRP on the ATP-induced release of IL-1β by LPS-primed U937 cells, suggesting that at least one NOS isoform is involved in signaling. We used the general NOS inhibitors L-NAME (0.1 – 10 µM) (60–62) and L-NIO (0.5 – 50 µM) (63) as well as 1400W (0.01 – 1 µM), which more specifically inhibits iNOS (64) and N-PLA (0.1 – 10 µM), which is regarded as an inhibitor of nNOS (35). In the same line, L-NIO and L-NAME provoked similar effects in THP-1 cells, another monocytic cell line, and in THP-1 cell-derived macrophages. From these inhibitor studies it is, however, difficult to conclude, which NOS isoforms are involved. As iNOS is constitutively active and mainly regulated by its protein expression (36, 37), a swift iNOS mediated inhibition of the P2X7R is not likely. This NOS isoform is expressed by activated pro-inflammatory monocytes and macrophages and plays a decisive role in host defense against infections (36). We decided to further investigate whether eNOS plays an essential role in signaling, because its mRNA was present in LPS-primed monocytic U937 cells, while the mRNA of nNOS was hardly detectable. In line with this assumption, the down-modulation of eNOS expression by siRNA in U937 cells blunted the inhibitory effect of nAChR agonists on the ATP-induced release of IL-1β, while transfection of a control siRNA had no such effect. This was convincingly confirmed by using PBMCs from wild-type and eNOS gene-deficient mice. While in wild-type mice an effective inhibition of the ATP-mediated IL-1β by nAChR agonists was seen, this effect was abolished in PBMCs from eNOS gene-deficient mice. Although compensatory up- or down-regulation of other genes has been described for gene-deficient mice in general, the complete absence the cholinergic inhibition of IL-1β release in eNOS gene-deficient cells is remarkable. We conclude, that eNOS plays an essential role in the inhibition of ATP-induced IL-1β release by nAChR stimulation in monocytic cells. The mechanism of how the function of eNOS is activated by stimulation of nAChRs is still unclear and deserves further research. Further, we cannot exclude an additional involvement of nNOS or iNOS in the regulation of P2X7R function.

During the process of activation, eNOS is mainly recruited to membrane domains, where NO is produced locally from its substrate arginine in the presence of the cofactor tetrahydrobiopterin (65–68). NO is a very reactive compound with a biological half-life below one second and thus most likely reacts with targets in its direct vicinity (41). NO and reactive oxygen species, which are often produced by activated mononuclear phagocytes (69), can react to form peroxynitrite, another highly reactive compound (40). Two different pathways of NO signaling have been described. In neurotransmission and vasodilation the soluble guanylyl cyclase is activated, which promotes the formation of the second messenger cyclic guanylyl monophosphate (41). Another pathway, which is most probably involved in the inhibition of the ionotropic function of the P2X7R, involves posttranslational S-nitrosylation at cysteine residues of proteins by NO or nitration by peroxynitrite, which both can modify protein structure and function (40, 41). While nitrosylation is a reversible modification, nitration is rather stable (40, 41).

To confirm the involvement of NO or NO-related compounds in the inhibition of the ATP-induced IL-1β release, we performed a series of experiments using the NO donors SNAP and SIN-1 (39). In all cell types investigated, which included monocytic U937 cells and THP-1 cells, primary human blood monocytes, THP-1-dependent macrophages, and murine BMDMs, SNAP and SIN-1 reduced the BzATP- or ATP-induced secretion of IL-1β. Depending on the cell type investigated, SNAP and SIN-1 fully inhibited or at least significantly blunted the ATP-induced release of IL-1β by LPS-primed monocytes or macrophages. As NO produced by iNOS was shown before to inhibit the activity of the inflammasome by nitrosylation of NLRP3 (70–72), the question remained if application of a NO- or a peroxynitrite donor inhibits the formation of active inflammasomes or interfered with the function of the P2X7R. Therefore, we included experiments, in which the pore-forming bacterial toxin nigericin was given as an ATP-independent stimulus for inflammasome activation. Apyrase was added in these experiments to exclude possible effects of endogenous ATP. In monocytic U937 cells, SNAP and SIN-1 had no effect on the nigericin-induced secretion of IL-1β, while in monocytic THP-1 cells, murine BMDMs and human monocytes a significant reduction of the secreted IL-1β was seen. In all cases, the effect of NO or NO-related compounds in the nigericin-induced IL-1β secretion was weaker compared to the effects on the ATP-induced secretion. These results suggest that, depending on the cell type, NO and peroxynitrite interferes with the function of the P2X7 receptor, but in addition they can interfere with other mechanisms such as the formation of inflammasomes that are shared with ATP-independent mechanisms of IL-1β maturation and release.

In different experimental settings, we showed that SIN-1 directly affects the ionotropic function of the ATP-sensitive P2X7R. TEVC measurements on *Xenopus laevis* oocytes heterologously expressing the human P2X7R is an accepted method to study its ionotropic function (73, 74). In line with our hypothesis, pre-incubation of the oocytes with SIN-1 significantly blunted the ATP-induced ion currents. In HEK cells over-expressing the human P2X7 receptor, ATP-induced P2X7R function was visualized by imaging of changes in intracellular Ca^2+^ concentrations and by whole cell patch-clamp measurements to monitor changes in transmembrane ion currents. Both, ATP-mediated changes in Ca^2+^ concentrations and ion current changes were indeed significantly blunted in the presence of SIN-1. We conclude from these results, that a NO-dependent modification of the P2X7R impairs its ionotropic function.

HEK cells over-expressing human P2X7R were also used to identify amino acids in the P2X7R C-terminus that might be modified by NO-related compounds. We provide evidence that a NO-dependent modification of the cysteine residue C377 plays an essential role in the inhibition of the ionotropic function of the P2X7R and suggest, that this residue is modified upon stimulation of nAChRs in human mononuclear phagocytes. When C377 is mutated to an alanine and expressed by HEK cells instead of the wildtype receptor, the NO and peroxynitrite donor SIN-1 does not inhibit the ionotropic function of the P2X7R anymore. C377 is the last cysteine out of six cysteine residues that form the cysteine-rich domain of the cytoplasmic C-terminus of the human P2X7R (9, 47). When palmitoylated, the cysteine-rich domains form a membrane anchor, which prevents early receptor desensitization, a typical feature of other ATP receptors such as the P2X3R lacking this cysteine-rich domain (9). Interestingly, cysteine nitrosylation was shown before as a mechanism regulating protein function and intracellular localization by competing with protein fatty acylation (54–57). Acylation at C377 does not seem to be essential for the ionotropic function of the P2X7R, because the mutation of the cysteine at position C377 to an alanine did not prevent P2X7R function. A replacement of C377 by the small and non-polar alanine does not perturb the membrane anchor, which contains five other cysteines that can by palmitoylated that might be functionally sufficient. However, it is conceivable that a polar NO-dependent modification at C377, which is in close contact to the transmembrane region of the P2X7R causes conformational changes that impair ion channel function, while a mutation of C377 to the small lipophilic alanine does not (9).

We further mutated C388 to alanine and expressed the mutant receptor in HEK cells. A cysteine located in a similar amino acid sequence was identified before as a putative nitrosylation site of the NMDA receptor that down-modulates its ionotropic function (10). This cysteine is located close to the region that interacts with the N-terminus in the cytoplasmic cap, which enables ion channel function of the P2X7R (9). The results of our experiments were, however, ambiguous. The ionotropic response of the C388A-P2X7R mutant was unimpaired as was that of the C377A mutant. However, the behavior of the C388A mutant in the presence of SIN-1 and ATP was highly variable ranging from a full inhibition of the ATP-induced ion currents to a more than twofold increase in the ion current changes. At present, we cannot explain these observations, but a mutation at C388 seems to destabilize the P2X7R, when NO donors are present.

Our study has numerous limitations. We did not touch the question, of how eNOS is activated in response to nAChR stimulation. This aspect needs to be investigated in future studies. It is also unclear, if iNOS and nNOS also essentially contribute to signaling, which might be suggested by the fact that N-PLA, a nNOS-specific NOS inhibitor, and 1400W, an iNOS-specific inhibitor, revert the effect of nAChR agonist on the ATP-mediated IL-1β release. As already discussed, we refrained from investigating nNOS and iNOS, but obviously more experiments are needed including experiments on blood cells from nNOS and iNOS gene-deficient mice. Further analyses are needed to directly show protein modification at C377 upon stimulation of nAChRs in mononuclear phagocytes and to directly identify the kind of modification. We also do not answer the question if other cysteines of the P2X7R are also involved in the cholinergic control mechanism. A comprehensive study of nAChR-induced protein modifications of the P2X7R would provide important insights into the functional regulation of the P2X7R.

## Conclusions

The conclusions that can be drawn from this study are summarized in **Figure 9**. We elucidated an essential part of the signaling mechanism that links the stimulation of monocytic nAChRs to the inhibition of the ionotropic function of the P2X7R in LPS-primed human and murine mononuclear phagocytes. Stimulation of nAChRs activates eNOS by a mechanism that still remains to be elucidated. We provide evidence, that an NO- or peroxynitrite-dependent modification of the P2X7R at C377 plays an essential role in the inhibition of its ionotropic function. C377 belongs to the cytoplasmic C-terminal tail of the P2X7R, which contains several cysteine residues that can be palmitoylated to form a membrane anchor. To the best of our knowledge, this is the first report on a site-specific NOS-mediated inhibition of the ionotropic function of the P2X7R. These results are of high medical interest, because P2X7R modification results in a striking reduction in the ATP-mediated release of monocytic IL-1β. Although more research is needed to elucidate this mechanism in every detail, we provide new insights into the regulation of the P2X7R, which might pave the way towards new therapeutics preventing over-shooting sterile inflammation.

## Data availability statement

The original contributions presented in the study are included in the article/**Supplementary Material**. Further inquiries can be directed to the corresponding author.

## Ethics statement

The studies involving human participants were reviewed and approved by ethics committee of the medical faculty Giessen, Germany (No. 90/18). The patients/participants provided their written informed consent to participate in this study. The animal study was reviewed and approved by University Düsseldorf, Germany, reference No. O16/04; Regierungspräsidium Giessen, Germany, reference no. 571_M. Written informed consent was obtained from the owners for the participation of their animals in this study.

## Author contributions

KR participated in the research design, performance of experiments, interpretation of the data, and writing of the manuscript. NA, VKS, SHY, MM and AZ participated in performance of the experiments, interpretation of the data, and editing of the manuscript. SW and SS participated in the performance of experiments. JL, AH, K-DS, MR, AG, WP, IM participated in the research design, interpretation of the data, and editing of the manuscript. GS and VG participated in the research design, interpretation of the data and writing of the manuscript. All authors contributed to the article and approved the submitted version.
