# Supplementary material for: Activation of endothelial NO synthase and P2X7 receptor modification mediates the cholinergic control of ATP-induced interleukin-1β release by mononuclear phagocytes
Source: Front Immunol. 2023 Mar 9;14:1140592. doi: 10.3389/fimmu.2023.1140592 (PMC10034071; doi:10.3389/fimmu.2023.1140592)
Supplement: Supplementary file 1 [file DataSheet_1.docx]

Supplementary Material

Activation of endothelial NO synthase and P2X7 receptor modification mediates the cholinergic control of ATP-induced interleukin-1β release by mononuclear phagocytes

**Katrin Richter^1,*^, NilayAsci^1^, Vijay K. Singh^2^, Sanaria Hawro Yakoob^3^, Marion Meixner^1^, Anna Zakrzewicz^1^, Juliane Liese^1^, Andreas Hecker^1^, Sigrid Wilker^1^, Sabine Stumpf^1^, Klaus-Dieter Schlüter^4^, Marius Rohde^2^, Axel Gödecke^5^, Winfried Padberg^1^, Ivan Manzini^6^, Günther Schmalzing^3^, Veronika Grau^1^**

*** Correspondence:**

Katrin Richter

Katrin.Richter@chiru.med.uni-giessen.de

# Supplementary Figures and Tables


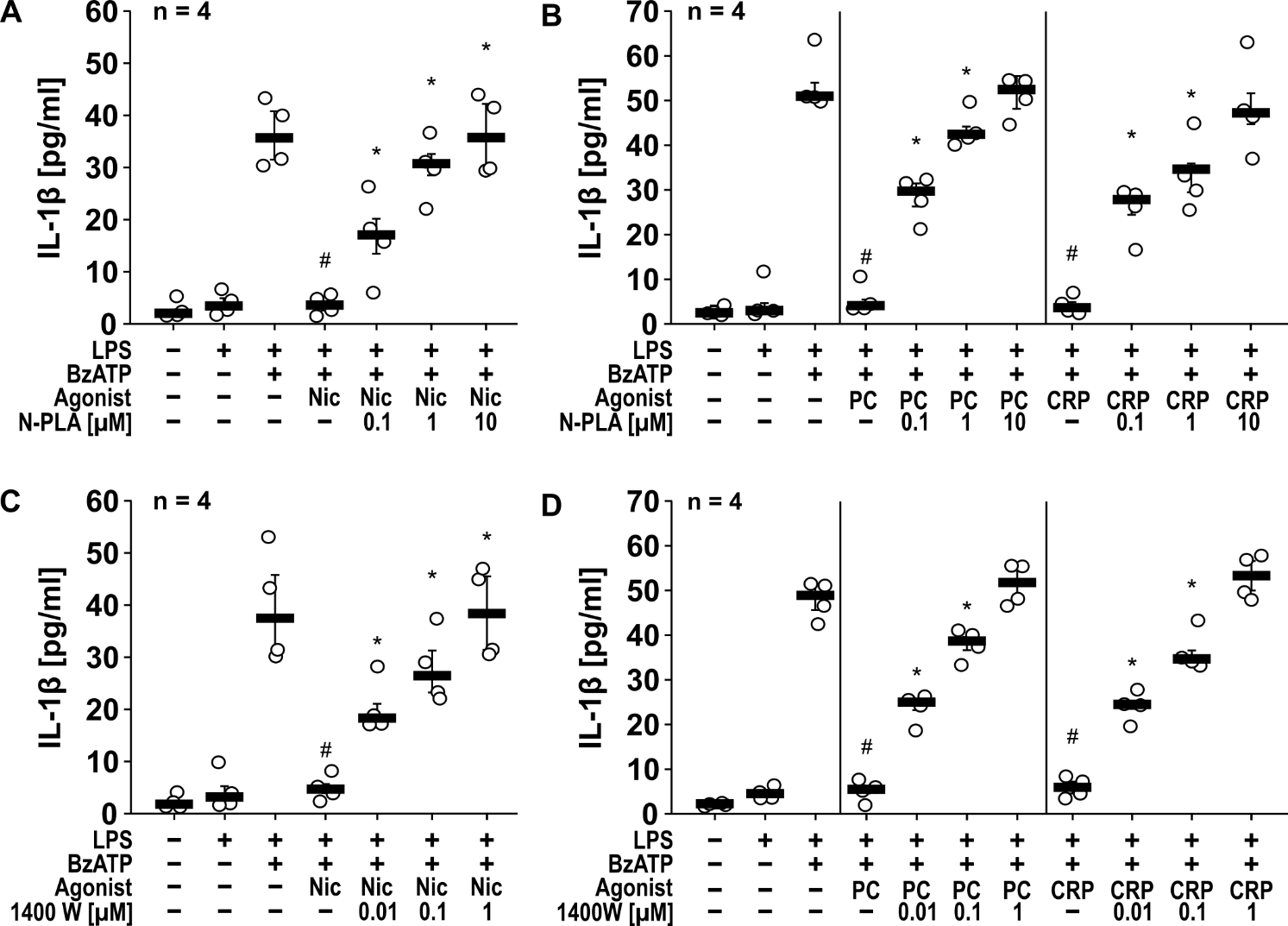


**Supplementary Figure S1: The inhibitory potential of nicotine (Nic), phosphocholine (PC) and C-reactive protein (CRP) on the BzATP-induced release of interleukin-1β (IL-1β) by human monocytic U937 cells is reversed by nitric oxide synthase (NOS) inhibitors.** U937 cells were primed with LPS (1 µg/ml) for 5 h, and BzATP (2’/3’-O-(4-benzoylbenzoyl)adenosine-5’-triphosphate, tri(triethylammonium) salt; 100 µM) was added for another 30 min to trigger IL-1β release, which was measured by ELISA. The inhibitory potential of the nicotinic acetylcholine receptor (nAChR) agonists Nic (100 µM), PC (100 µM) and CRP (5 µg/ml) on the BzATP-induced release of IL-1β was investigated in absence and presence of a panel of NOS inhibitors: A, B) N-PLA (N-omega-allyl-L-arginine hydrochloride) and C, D) 1400 W (1400 W dihydrochloride). The inhibitory potential of nAChR agonists on the BzATP-induced release was concentration-dependently reversed by all NOS inhibitors. Some IL-1β values (untreated, LPS, LPS+BzATP, LPS+BzATP+Nic) served as controls for multiple NOS inhibitors (Figure 1A, C). Data are presented as individual data points, bars represent median, whiskers percentiles 25 and 75. # p ≤ 0.05 signiﬁcantly different from samples, in which BzATP was given alone, * p ≤ 0.05 signiﬁcantly different from BzATP + nicotine. Kruskal-Wallis followed by Mann-Whitney rank sum test.

**
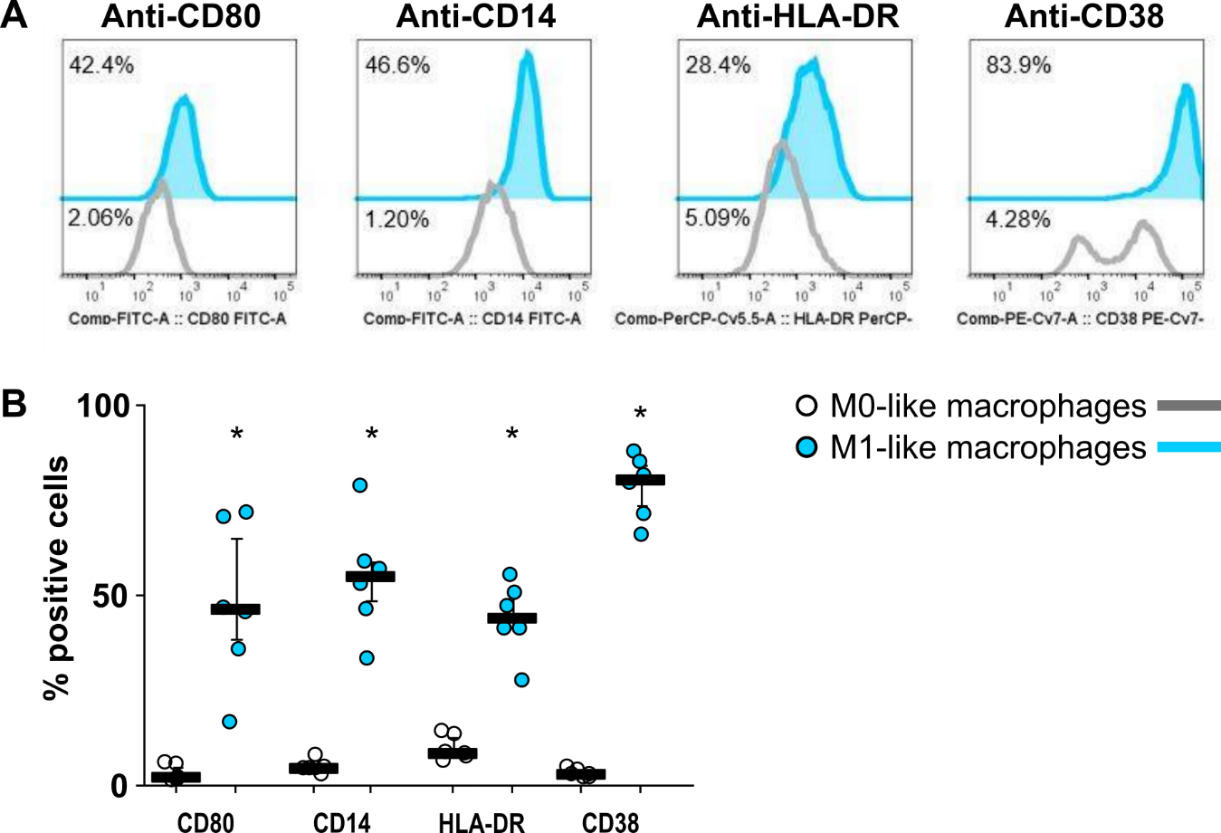
**

**Supplementary Figure S2: Flow cytometry analyses confirmed that differentiated THP-1 cells expressed cell surface markers typical for M1-like macrophages.** A) Representative histograms of CD80, CD14, HLA-DR and CD38 expression in M0- and M1-like THP-1 cell-derived macrophages. B) Quantification of macrophage subsets according to the cell surface marker expression in M0- and M1-like THP-1 cell-derived macrophages (n = 6). Data were analyzed by Kruskal-Wallis test followed by the Mann-Whitney rank sum test and presented as individual data points, bars represent median, whiskers percentiles 25 and 75. ∗ p ≤ 0.05 signiﬁcantly different from corresponding M0-like macrophages.

**
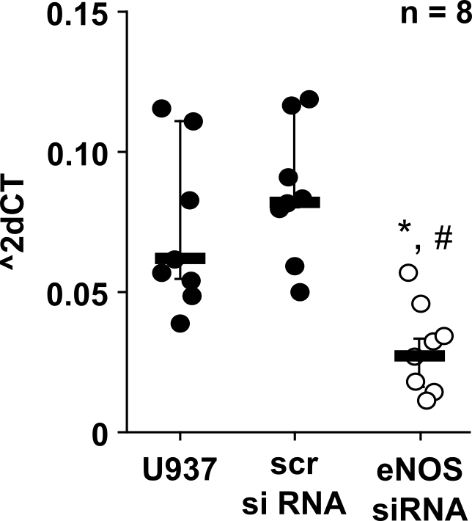
**

**Supplementary Figure S3: Reduction of mRNA expression of endothelial nitic oxide synthase (eNOS, *NOS3*) upon siRNA transfection.** U937 cells were left untreated (U937) or transfected with control siRNA or with siRNA speciﬁcally targeting eNOS (NOS3). Forty-eight hours after transfection, the mRNA expression of eNOS was analyzed by real-time RT-PCR. Transfection with gene-speciﬁc siRNA efﬁciently down-regulated the mRNA expression of NOS3. Data were analyzed by Kruskal-Wallis test followed by the Mann-Whitney rank sum test and presented as individual data points, bars represent median, whiskers percentiles 25 and 75. ∗ p ≤ 0.05 signiﬁcantly different from samples transfected with control siRNA. # p ≤ 0.05 signiﬁcantly different from untreated samples.

**
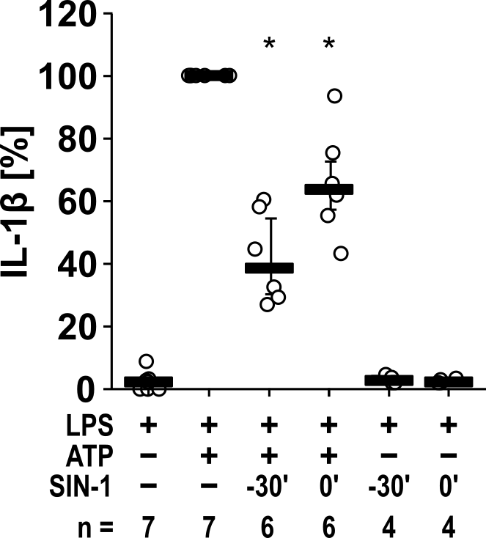
**

**Supplementary Figure S4: The ATP-induced release of interleukin (IL)-1β by mouse peripheral blood mononuclear cells (PBMCs) is inhibited by the NO donor SIN-1.** Freshly isolated mouse PBMCs from wild-type mice were left untreated or stimulated with ATP (1 mM) for 30 min, in the presence or absence of SIN-1. Addition of SIN-1 30 min before ATP (SIN-1 t = -30’) or shortly before ATP (SIN-1 t = 0’) inhibited the ATP-induced release of IL-1β. Data are presented as individual data points, bars represent median, whiskers percentiles 25 and 75. ∗ p ≤ 0.05 signiﬁcantly different from samples in which ATP was given alone. Friedman test followed by the Wilcoxon signed-rank test.

**
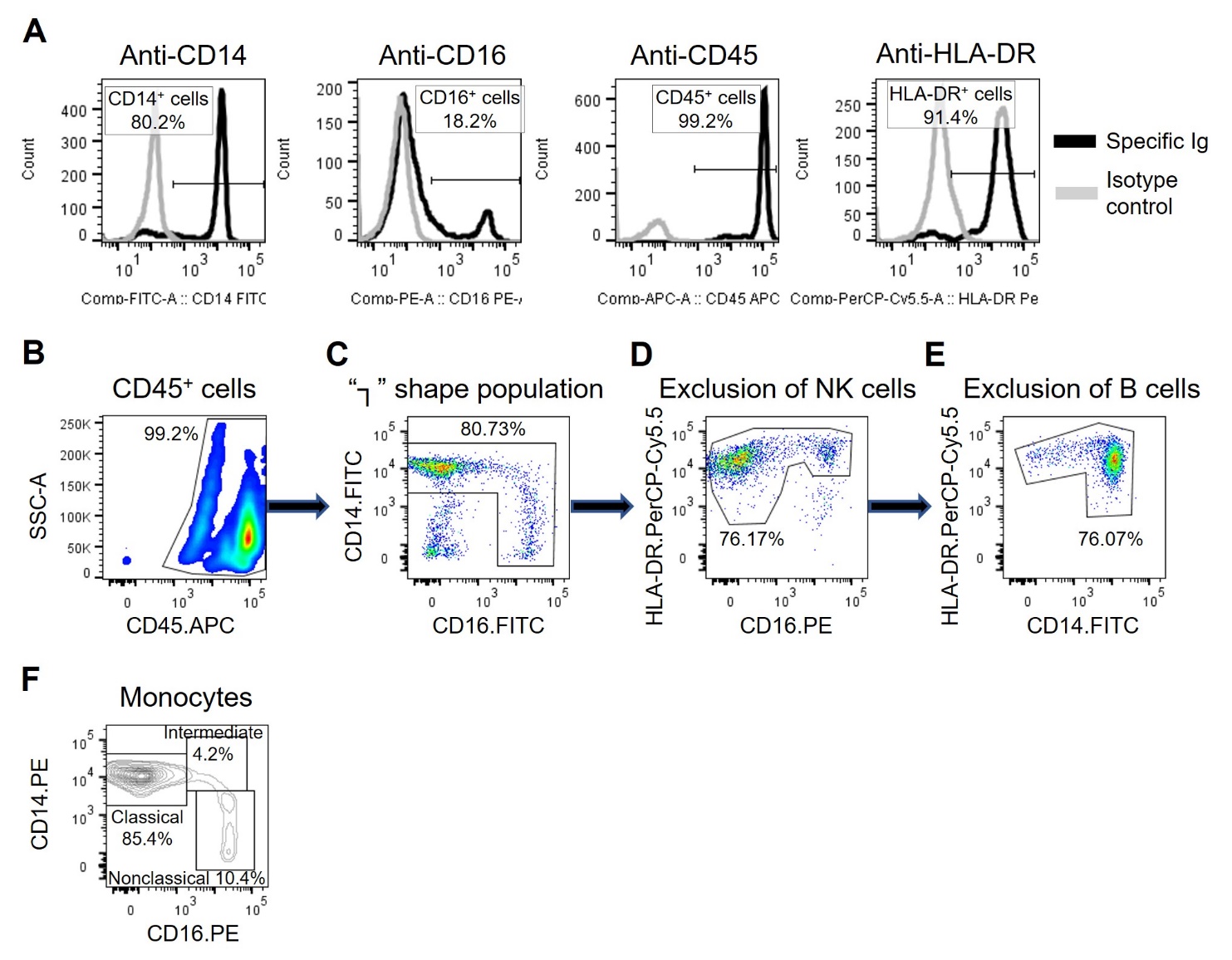
**

**Supplementary Figure S5: Gating strategy in flow cytometry analysis to test for the purity of RosetteSep^TM^ enriched human monocytes obtained from blood samples of one healthy volunteer.** A) Representative single cell surface marker histograms of CD14, CD16, HLA-DR and CD45 of one out of three experiments on RosetteSep^TM^ enriched human monocytes. B-F) Monocyte Gating strategy. B) Selection of blood immune cells based on their side scatter – area (SSC-A) vs. CD45 properties. (C) CD16 vs. CD14 plot: gating to select monocytes based on their characteristic "┐" shape. (D) CD16 vs. HLA-DR plot: gating to select HLA-DR positive cells and remove natural killer (NK) cells and neutrophils. (E) CD14 vs. HLA-DR: gating to exclude the B cells (HLA-DR high/CD14 low) from the monocytes. (F) Percentage of classical, intermediate, and non-classical monocyte subsets. Representative results of one out of three experiments.

**Supplementary Figure S6: Sequence Alignment of the rat (rP2X7) and human (hP2X7) P2X7 receptor.** Sequence alignment using Clustal Omega software (<https://www.ebi.ac.uk/Tools/msa/clustalo/>; DOI: 10.1093/nar/gkac240) revealed 80.17 % sequence identity. Two highly conserved regions are marked (yellow). The highlighted region in red denotes the conserved active site residues of cysteine C377 and C388.

**Table S1: Cell death in U937 and THP-1 cells, human or mouse peripheral blood mononuclear cells (hPBMCs or mPBMCs) as estimated by the lactate dehydrogenase (LDH) activity in cell culture supernatants.**

| **Figure** | **Cells, treatment** | **Cell death [%]**  **mean ± SD** | **n** |
| --- | --- | --- | --- |
| **Figure 1 A, C, G, E** | U937, - | 3.6 ± 1.9 | 9 |
|  | U937, LPS | 3.9 ± 2.3 | 9 |
|  | U937, LPS, BzATP | 4.1 ± 0.8 | 9 |
|  | U937, LPS, BzATP, Nic 100 µM | 3.7 ± 1.5 | 9 |
|  | U937, LPS, BzATP, Nic 100 µM, L-NIO 0.5 µM | 4.5 ± 1.7 | 4 |
|  | U937, LPS, BzATP, Nic 100 µM, L-NIO 5 µM | 3.5 ± 0.6 | 4 |
|  | U937, LPS, BzATP, Nic 100 µM, L-NIO 50 µM | 5.0 ± 1.4 | 4 |
|  | U937, LPS, BzATP, Nic 100 µM, N-PLA 0.1 µM | 4.5 ± 1.0 | 4 |
|  | U937, LPS, BzATP, Nic 100 µM, N-PLA 1 µM | 6.3 ± 1.9 | 4 |
|  | U937, LPS, BzATP, Nic 100 µM, N-PLA 10 µM | 4.8 ± 1.5 | 4 |
|  | U937, LPS, BzATP, Nic 100 µM, 1400 W 0.01 µM | 4.8 ± 1.3 | 4 |
|  | U937, LPS, BzATP, Nic 100 µM, 1400 W 0.1 µM | 4.3 ± 1.0 | 4 |
|  | U937, LPS, BzATP, Nic 100 µM, 1400 W 1 µM | 3.5 ± 0.6 | 4 |
|  | U937, LPS, BzATP, Nic 100 µM, L-NAME 0.1 µM | 3.3 ± 1.3 | 5 |
|  | U937, LPS, BzATP, Nic 100 µM, L-NAME 1 µM | 3.3 ± 0.5 | 5 |
|  | U937, LPS, BzATP, Nic 100 µM, L-NAME 10 µM | 3.8 ± 1.8 | 5 |
| **Figure 1 B, D, F, H** | U937, - | 1.7 ± 0.9 | 16 |
|  | U937, LPS | 1.4 ± 1.0 | 16 |
|  | U937, LPS, BzATP | 1.6 ± 1.2 | 16 |
|  | U937, LPS, BzATP, PC 100 µM | 1.7 ± 0.8 | 16 |
|  | U937, LPS, BzATP, PC 100 µM, L-NIO 0.5 µM | 1.0 ± 0.4 | 4 |
|  | U937, LPS, BzATP, PC 100 µM, L-NIO 5 µM | 0.9 ± 0.5 | 4 |
|  | U937, LPS, BzATP, PC 100 µM, L-NIO 50 µM | 1.2 ± 0.6 | 4 |
|  | U937, LPS, BzATP, PC 100 µM, N-PLA 0.1 µM | 1.8 ± 1.5 | 4 |
|  | U937, LPS, BzATP, PC 100 µM, N-PLA 1 µM | 2.1 ± 0.6 | 4 |
|  | U937, LPS, BzATP, PC 100 µM, N-PLA 10 µM | 1.7 ± 0.9 | 4 |
|  | U937, LPS, BzATP, PC 100 µM, 1400 W 0.01 µM | 1.3 ± 1.1 | 4 |
|  | U937, LPS, BzATP, PC 100 µM, 1400 W 0.1 µM | 0.9 ± 0.9 | 4 |
|  | U937, LPS, BzATP, PC 100 µM, 1400 W 1 µM | 0.9 ± 0.8 | 4 |
|  | U937, LPS, BzATP, PC 100 µM, L-NAME 0.1 µM | 1.9 ± 1.2 | 4 |
|  | U937, LPS, BzATP, PC 100 µM, L-NAME 1 µM | 2.5 ± 1.3 | 4 |
|  | U937, LPS, BzATP, PC 100 µM, L-NAME 10 µM | 1.4 ± 0.4 | 4 |
|  | U937, LPS, BzATP, CRP 5 µg/ml | 1.2 ± 0.8 | 16 |
|  | U937, LPS, BzATP, CRP 5 µg/ml, L-NIO 0.5 µM | 1.0 ± 0.3 | 4 |
|  | U937, LPS, BzATP, CRP 5 µg/ml, L-NIO 5 µM | 1.5 ± 0.7 | 4 |
|  | U937, LPS, BzATP, CRP 5 µg/ml, L-NIO 50 µM | 1.0 ± 0.9 | 4 |
|  | U937, LPS, BzATP, CRP 5 µg/ml, N-PLA 0.1 µM | 1.3 ± 1.0 | 4 |
|  | U937, LPS, BzATP, CRP 5 µg/ml, N-PLA 1 µM | 1.7 ± 1.1 | 4 |
|  | U937, LPS, BzATP, CRP 5 µg/ml, N-PLA 10 µM | 1.6 ± 1.1 | 4 |
|  | U937, LPS, BzATP, CRP 5 µg/ml, 1400 W 0.01 µM | 0.8 ± 0.8 | 4 |
|  | U937, LPS, BzATP, CRP 5 µg/ml, 1400 W 0.1 µM | 1.0 ± 1.1 | 4 |
|  | U937, LPS, BzATP, CRP 5 µg/ml, 1400 W 1 µM | 1.1 ± 0.5 | 4 |
|  | U937, LPS, BzATP, CRP 5 µg/ml, L-NAME 0.1 µM | 2.1 ± 0.4 | 4 |
|  | U937, LPS, BzATP, CRP 5 µg/ml, L-NAME 1 µM | 2.3 ± 0.4 | 4 |
|  | U937, LPS, BzATP, CRP 5 µg/ml, L-NAME 10 µM | 2.5 ± 0.7 | 4 |
| **Figure 2** | THP-1, - | 7.3 ± 1.5 | 6 |
|  | THP-1, LPS | 7.5 ± 1.0 | 6 |
|  | THP-1, LPS, BzATP | 10.4 ± 1.7 | 6 |
|  | THP-1, LPS, BzATP, Nic 100 µM | 8.8 ± 1.9 | 6 |
|  | THP-1, LPS, BzATP, Nic 100 µM, L-NIO 50 µM | 10.7 ± 2.7 | 6 |
|  | THP-1, LPS, BzATP, Nic 100 µM, L-NAME 10 µM | 10.2 ± 2.5 | 6 |
|  | THP-1, LPS, BzATP, PC 200 µM | 8.2 ± 1.5 | 6 |
|  | THP-1, LPS, BzATP, PC 200 µM, L-NIO 50 µM | 9.8 ± 2.8 | 6 |
|  | THP-1, LPS, BzATP, PC 200 µM, L-NAME 10 µM | 10.7 ± 1.9 | 6 |
|  | THP-1, LPS, BzATP, CRP 10 µg/ml | 8.2 ± 1.3 | 6 |
|  | THP-1, LPS, BzATP, CRP 10 µg/ml, L-NIO 50 µM | 9.7 ± 2.7 | 6 |
|  | THP-1, LPS, BzATP, CRP 10 µg/ml, L-NAME 10 µM | 10.0 ± 2.8 | 6 |
|  | THP-1, LPS, BzATP, L-NIO 50 µM | 9.3 ± 2.3 | 6 |
|  | THP-1, LPS, BzATP, L-NAME 10 µM | 9.7 ± 2.3 | 6 |
| **Figure 3A** | U937 control siRNA, LPS | 4.4 ± 3.0 | 11 |
|  | U937 control siRNA, LPS, BzATP | 3.6 ± 2.2 | 11 |
|  | U937 control siRNA, LPS, BzATP, Nic 100 µM | 4.1 ± 2.2 | 8 |
|  | U937 control siRNA, LPS, BzATP, PC 100 µM | 4.0 ± 0.8 | 4 |
|  | U937 control siRNA, LPS, BzATP, CRP 5 µg/ml | 5.8 ± 3.8 | 4 |
|  | U937 eNOS siRNA, LPS | 5.5 ± 3.5 | 11 |
|  | U937 eNOS siRNA, LPS, BzATP | 4.9 ± 3.0 | 11 |
|  | U937 eNOS siRNA, LPS, BzATP, Nic 100 µM | 4.8 ± 1.6 | 8 |
|  | U937 eNOS siRNA, LPS, BzATP, PC 100 µM | 4.0 ± 0.8 | 4 |
|  | U937 eNOS siRNA, LPS, BzATP, CRP 5 µg/ml | 6.5 ± 3.0 | 4 |
| **Figure 3B** | eNOS^+/+^ mice, - | 2.3 ± 1.4 | 8 |
|  | eNOS^+/+^ mice, BzATP | 2.5 ± 1.7 | 8 |
|  | eNOS^+/+^ mice, BzATP, Nic 100 µM | 1.8 ± 2.1 | 4 |
|  | eNOS^+/+^ mice, BzATP, PC 100 µM | 2.4 ± 1.8 | 4 |
|  | eNOS^+/+^ mice, BzATP, Cho 100 µM | 1.7 ± 2.1 | 4 |
|  | eNOS^+/+^ mice, BzATP, ACh 10 µM | 1.7 ± 1.7 | 4 |
|  | eNOS^-/-^ mice, - | 8.1 ± 4.0 | 8 |
|  | eNOS^-/-^ mice, BzATP | 10.9 ± 4.6 | 8 |
|  | eNOS^-/-^ mice, BzATP, Nic 100 µM | 7.3 ± 3.4 | 6 |
|  | eNOS^-/-^ mice, BzATP, PC 100 µM | 8.4 ± 3.0 | 6 |
|  | eNOS^-/-^ mice, BzATP, Cho 100 µM | 7.4 ± 2.7 | 6 |
|  | eNOS^-/-^ mice, BzATP, ACh 10 µM | 7.6 ± 2.7 | 6 |
| **Figure 4 A-C** | U937, - | 2.4 ± 2.2 | 12 |
|  | U937, LPS | 3.0 ± 2.7 | 8 |
|  | U937, LPS, BzATP ± solvent | 2.3 ± 1.6 | 8 |
|  | U937, LPS, BzATP, Nic 100 µM | 3.0 ± 2.9 | 4 |
|  | U937, LPS, BzATP, SNAP 0.01 mM | 1.3 ± 1.0 | 4 |
|  | U937, LPS, BzATP, SNAP 0.1 mM | 2.0 ± 1.6 | 4 |
|  | U937, LPS, BzATP, SNAP 1 mM | 2.3 ± 1.7 | 4 |
|  | U937, LPS, BzATP, SIN-1 -30‘ | 1.3 ± 0.7 | 4 |
|  | U937, LPS, BzATP, SIN-1 0‘ | 0.7 ± 0.6 | 4 |
|  | U937, LPS, Nig ± solvent | 0.8 ± 0.3 | 4 |
|  | U937, LPS, Nig, SIN-1 1 mM -30‘ | 2.7 ± 0.7 | 4 |
|  | U937, LPS, Nig, SIN-1 0‘ | 2.9 ± 0.6 | 4 |
| **Figure 4 D-E** | THP-1, - | 12.1 ± 5.5 | 15 |
|  | THP-1, LPS | 11.6 ± 4.1 | 14 |
|  | THP-1, LPS, BzATP ± solvent | 11.3 ± 5.4 | 9 |
|  | THP-1, LPS, BzATP, ACh 10 µM | 9.2 ± 4.5 | 6 |
|  | THP-1, LPS, BzATP, SNAP 10 mM | 6.2 ± 3.5 | 6 |
|  | THP-1, LPS, BzATP, SIN-1 -30‘ | 5.3 ± 2.7 | 6 |
|  | THP-1, LPS, BzATP, SIN-1 -20‘ | 6.0 ± 2.7 | 6 |
|  | THP-1, LPS, BzATP, SIN-1 -10‘ | 7.8 ± 3.5 | 6 |
|  | THP-1, LPS, BzATP, SIN-1 0‘ | 6.2 ± 2.2 | 6 |
|  | THP-1, LPS, SNAP 10 mM | 8.0 ± 4.1 | 6 |
|  | THP-1, LPS, SIN-1 -30‘ | 9.3 ± 4.5 | 6 |
|  | THP-1, LPS, SIN-1 0‘ | 8.3 ± 4.2 | 3 |
|  | THP-1, Nig | 19.7 ± 3.1 | 3 |
|  | THP-1, LPS, Nig ± solvent | 18.3 ± 4.9 | 6 |
|  | THP-1, LPS, Nig, SNAP 10 mM | 37.5 ± 12.7 | 6 |
|  | THP-1, LPS, Nig, SIN-1 -30‘ | 15.3 ± 4.8 | 6 |
|  | THP-1, LPS, Nig, SIN-1 -20‘ | 13.8 ± 4.2 | 6 |
|  | THP-1, LPS, Nig, SIN-1 -10‘ | 15.0 ± 4.9 | 6 |
|  | THP-1, LPS, Nig, SIN-1 0‘ | 17.5 ± 4.7 | 6 |
| **Figure 5 A-D** | Mouse BMDMs, - | 14.9 ± 4.4 | 13 |
|  | Mouse BMDMs, LPS | 16.7 ± 5.5 | 13 |
|  | Mouse BMDMs, LPS, ATP ± solvent | 18.2 ± 4.0 | 24 |
|  | Mouse BMDMs, LPS, ATP, SNAP 10 mM -30’ | 16.9 ± 6.8 | 11 |
|  | Mouse BMDMs, LPS, ATP, SNAP 10 mM 0’ | 14.5 ± 6.2 | 8 |
|  | Mouse BMDMs, LPS, SNAP 10 mM -30’ | 16.8 ± 2.6 | 6 |
|  | Mouse BMDMs, LPS, SNAP 10 mM 0’ | 13.8 ± 11.9 | 6 |
|  | Mouse BMDMs, LPS, ATP, SIN-1 -30’ | 14.5 ± 6.3 | 8 |
|  | Mouse BMDMs, LPS, ATP, SIN-1 0’ | 15.6 ± 3.9 | 8 |
|  | Mouse BMDMs, LPS, SIN-1 -30’ | 16.6 ± 7.4 | 8 |
|  | Mouse BMDMs, LPS, SIN-1 0’ | 17.1 ± 10.3 | 8 |
|  | Mouse BMDMs, Nig | 1.2 ± 2.2 | 11 |
|  | Mouse BMDMs, LPS, Nig ± solvent | 13.9 ± 11.7 | 22 |
|  | Mouse BMDMs, LPS, Nig, SNAP 10 mM -30’ | 13.2 ± 5.9 | 11 |
|  | Mouse BMDMs, LPS, Nig, SNAP 10 mM 0’ | 16.3 ± 9.8 | 8 |
|  | Mouse BMDMs, LPS, Nig, SIN-1-30’ | 3.0 ± 4.8 | 4 |
|  | Mouse BMDMs, LPS, Nig, SIN-1 0’ | 3.5 ± 4.7 | 6 |
| **Figure 5 E-H** | Human monoctyes, LPS | 3.3 ± 3.2 | 13 |
|  | Human monoctyes, LPS, BzATP ± solvent | 4.4 ± 3.5 | 24 |
|  | Human monoctyes, LPS, BzATP, SNAP 10 mM -30’ | 5.2 ± 2.5 | 6 |
|  | Human monoctyes, LPS, BzATP, SNAP 10 mM 0’ | 3.6 ± 2.0 | 7 |
|  | Human monoctyes, LPS, SNAP 10 mM-30’ | 4.6 ± 2.1 | 6 |
|  | Human monocytes, LPS, SNAP 10 mM0’ | 11.2 ± 9.9 | 9 |
|  | Human monoctyes, LPS, BzATP, SIN-1 -30’ | 8.0 ± 5.7 | 8 |
|  | Human monoctyes, LPS, BzATP, SIN-1 0’ | 6.8 ± 5.2 | 6 |
|  | Human monoctyes, LPS, SIN-1 -30’ | 5.0 ± 1.9 | 7 |
|  | Human monoctyes, LPS, SIN-1 0’ | 10.3 ± 12.3 | 7 |
|  | Human monoctyes, LPS, Nig ± solvent | 12.2 ± 8.2 | 18 |
|  | Human monoctyes, LPS, Nig, SNAP 10 mM -30’ | 12.9 ± 12.7 | 7 |
|  | Human monoctyes, LPS, Nig, SNAP 10 mM 0’ | 25.6 ± 19.1 | 11 |
|  | Human monoctyes, LPS, Nig, SIN-1 -30’ | 19.3 ± 11.3 | 8 |
|  | Human monoctyes, LPS, Nig, SIN-1 0’ | 19.0 ± 9.6 | 7 |
| **Figure S3** | Mouse PBMCs, LPS | 7.1 ± 9.3 | 7 |
|  | Mouse PBMCs, LPS, ATP | 12.1 ± 7.3 | 7 |
|  | Mouse PBMCs, LPS, ATP, SIN-1 -30’ | 2.2 ± 1.7 | 6 |
|  | Mouse PBMCs, LPS, ATP, SIN-1 0’ | 5.0 ± 3.3 | 6 |
|  | Mouse PBMCs, LPS, SIN-1 -30’ | 3.8 ± 5.0 | 4 |
|  | Mouse PBMCs, LPS, SIN-1 0’ | 6.5 ± 7.5 | 4 |

Cell death was estimated via measurement of the release of lactate dehydrogenase (LDH) into the cell culture medium. The data depicted in this table correspond to the experiments shown in the respective figures of the main part of this manuscript. Human monocytic U937 and THP-1 cells as well as mouse bone marrow-derived macrophages (BMDMs) were primed with lipopolysaccharide (LPS, 1 µg/ml, for 5 h) and further stimulated with 2´(3´)-O-(4-benzoylbenzoyl)adenosine 5´-triphosphate triethylammonium salt (BzATP; 100 mM); nigericin (Nig; 25 – 50 µM) or ATP (1 – 2 mM). Human monocytes were pulsed with LPS (5 ng/ml) for 25 min during the first washing step of isolation. Freshly isolated peripheral blood mononuclear leukocytes (PBMCs) obtained from wild-type mice (endothelial NO synthase, eNOS^+/+^) and mice deﬁcient in *Nos3* (eNOS^-/-^) were cultured for 2 h before BzATP (100 μM) or ATP (1 mM) was added for another 30 min. When appropriate, control experiments were performed with the corresponding concentrations of solvents (dimethyl sulfoxide/ethanol) without drugs. The concentration of diverse compounds is indicated in the table. ACh, acetylcholine; Cho, choline; Nic, nicotine; L-NAME, Nω-Nitro-L-arginine methyl ester; N-PLA, Nω-propyl-L-arginine; PC, phosphocholine; SIN-1, 1 mM; SNAP, S-nitroso-N-acetyl-DL-penicillamine.

**Supplementary Table S2: Antibodies used for flow cytometry analyses of THP-1 cell-derived macrophages and RosetteSep^TM^ enriched human monocytes.**

|  | **Marker** | **Fluorochrome** | **Clone** | **Company** |
| --- | --- | --- | --- | --- |
| Marked | Human CD3 | FITC | SK7 | Becton Dickinson |
|  | Human CD14 | FITC | M5E2 | BD Pharmingen |
|  | Human CD16 | PE | 3G8 | Becton Dickinson |
|  | Human CD19 | PE | 4G7 | Becton Dickinson |
|  | Human CD38 | PE-Cy7 | HIT2 | BioLegend |
|  | Human CD45 | APC | HI30 | BD Pharmingen |
|  | Human CD80 | FITC | BB1 | BD Pharmingen |
|  | Human HLA-DR | PerCP-Cy5.5 | L243 | BD Biosciences |
| Isotype | Mouse-IgG1, κ | APC | M1-14012 | eBioscience |
|  | Mouse-IgG1, κ | PerCP-Cy5 | 679.1Mc7 | Becton Dickinson |
|  | Mouse-IgG1, κ | PE | X40 | BD Biosciences |
|  | Mouse-IgG1, κ | FITC | X40 | BD Biosciences |
|  | Mouse IgG1, κ | PE-Cy7 | X40 | BD Biosciences |
|  | Mouse-IgG2a κ | FITC | X39 | BD Biosciences |
|  | Mouse-IgG2a, κ | PE | G155-178 | BD Pharmingen |
|  | Mouse-IgG2b, κ | PE | MPC-11 | BD Pharmingen |

**
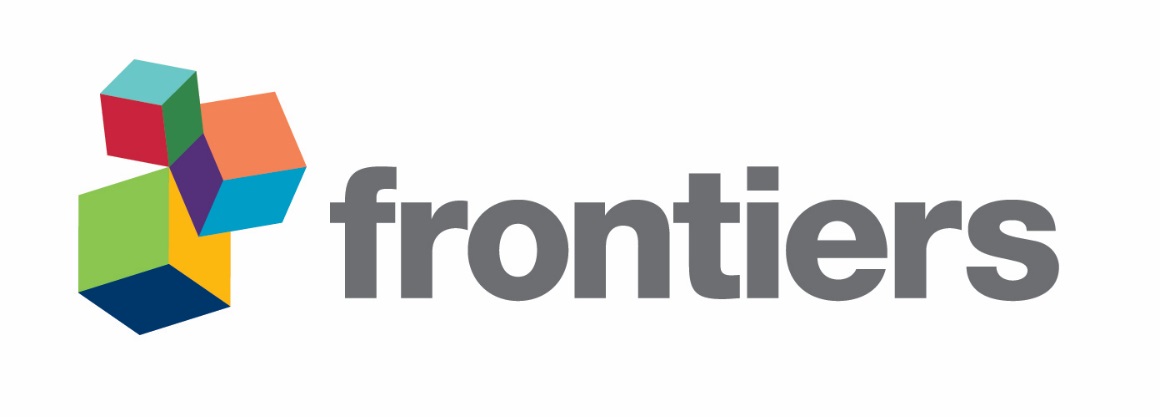
**
